# Coherence and fidelity aware routing in quantum networks

**DOI:** 10.1038/s41598-026-65444-1

**Published:** 2026-08-06

**Authors:** Hilal Sultan Duranoglu Tunc, Joy Halder, Azita Hajizade, Andreas Voigt, Bassem Arar, Riccardo Bassoli, Gerhard P. Fettweis, Frank H. P. Fitzek

**Affiliations:** 1 Deutsche Telekom Chair of Communication Networks, Technische Universität Dresden, Dresden, Germany; 2 Quantum Communication Networks Research Group, Technische Universität Dresden, Dresden, Germany; 3 Vodafone Chair Mobile Communications Systems, Technische Universität Dresden, Dresden, Germany; 4 Centre for Tactile Internet with Human-in-the-Loop, Technische Universität Dresden, Dresden, Germany

**Keywords:** Mathematics and computing, Physics

## Abstract

In quantum networks, routing mechanisms designed to deliver successfully multiple simultaneous requests to the destination under limited channel capacity should simultaneously consider resource usage optimization, entanglement-generation performance, and the quality of the end-to-end delivered entangled states. The routing approaches developed for quantum networks in the literature mostly consider the change in fidelity during routing. However, it should be noted that quantum states with the same fidelity value may have different levels of coherence. In this study, for the first time, a novel routing and purification approach for quantum networks is presented, using the end-to-end (E2E) relative entropy of coherence (REC) together with E2E fidelity to determine the purification level and the feasibility of candidate paths. In our study, the coherence- and fidelity-aware routing algorithm (CAFARA) is proposed. In CAFARA, the BBPSSW purification levels are determined for each candidate path and ordered from the lowest to the highest. This information is stored in a lookup table. Then, the lowest BBPSSW purification level that simultaneously satisfies the requested fidelity, REC, latency, and capacity constraints is selected. The E2E fidelity, REC, and required number of raw Bell pairs mentioned here are all stored together in the previously mentioned lookup table. This lookup table was constructed using imperfect initial Werner states, one-sided amplitude damping, Werner-state twirling, BBPSSW purification, and density-matrix-based entanglement swapping, and all calculations were completed before the routing process. As the final step, CAFARA selects the best path with the highest entanglement generation rate (EGR) from the selected feasible paths. In our study, the FARA-PostREC and FARA-NoREC algorithms were developed for comparison with CAFARA. While the FARA-PostREC algorithm uses the REC constraint during the final validation stage of the request, FARA-NoREC does not use any REC constraint; it uses only fidelity as the quality parameter of the paths and as the parameter for determining the purification level. In our simulations under varying link distance, channel capacity, network size, and request load, CAFARA achieved a better average request success rate than FARA-PostREC by reducing late-stage request drops related to coherence because it also uses REC during the purification-level determination stage while maintaining the required E2E fidelity and REC values. Although FARA-NoREC often accepts more requests and assumes that they are successfully delivered, the average final fidelity (AFF) and average final coherence (AFC) values of the requests considered successful are lower than those of the other algorithms; the AFC does not even satisfy the REC threshold. Overall, CAFARA provides a balanced trade-off between the quality-guaranteed request success rate, latency, purification overhead, and resource consumption.

## Introduction

Quantum networks, formed by interconnecting remote quantum nodes, are envisioned to scale globally^1–4^. Realizing this large-scale vision brings numerous complex challenges, such as routing protocol design, entanglement distribution, and the optimization of physical components. These challenges have been addressed from several perspectives in the literature, with various studies proposing solutions for quantum-network design, routing, and resource management^5–9^. The large-scale vision of the quantum internet requires the ability to efficiently enable communication between multiple source-destination (S-D) pairs that may be far apart, using quantum repeaters to establish multi-hop entanglement connections. Achieving this goal necessitates an efficient and reliable routing algorithm.

Quantum entanglement serves as a fundamental resource for quantum-network applications by enabling nonclassical correlations between distant parties^10^, while the no-cloning theorem imposes a fundamental constraint on the replication of unknown quantum states^11^. Coherence, which is a necessary condition for the existence of entanglement, is the ability of a quantum system to preserve the phase information belonging to its superposition state^12^. If coherence is lost, the qubit can no longer carry quantum information, entanglement is lost, and fidelity decreases.

Previous studies have investigated the relation between coherence, decoherence, and fidelity from different perspectives. Prakash et al. examined how decoherence affects teleportation fidelity when entangled coherent states are used as the teleportation resource^13^, while Liu et al. introduced a fidelity-based coherence measure satisfying the standard requirements of coherence quantification^14^. Mokeev et al. modeled decoherence during the transport of entangled qubits and showed how correlated noise influences transport fidelity^15^, and Guo et al. derived the effect of correlated amplitude-damping, phase-damping, and depolarizing channels on quantum teleportation fidelity^16^. Building on this line of work, Tunc et al. related the end-to-end (E2E) fidelity of a quantum-network routing path to the E2E REC under different noise models^17,18^.

Fidelity and REC are not two reliability metrics that replace each other, but two reliability metrics that complement each other. E2E fidelity measures how close the distributed quantum state is to the targeted entangled state. In contrast, REC shows how well this state preserves its usable quantum superposition structure. Therefore, a routing decision made only by considering fidelity may be incomplete in some cases. This is because, although a path may appear to satisfy the fidelity threshold, the coherence level on that path may not be sufficient for reliable quantum information processing. This distinction is especially important under realistic noise models. This is because different noise mechanisms do not necessarily produce the same effect on fidelity and coherence. In other words, two paths with similar E2E fidelity values may have different REC values^17,18^. In this case, while a fidelity-only algorithm may see these two paths as having similar quality, an REC and fidelity-aware algorithm can distinguish the path that is more reliable.

Adding REC as a reliability measure provides three main gains. First, it prevents the selection of paths whose fidelity value is sufficient but whose coherence level is low. Second, it allows the purification target to be made stricter only when necessary, that is, when the REC constraint requires it. Third, it ensures that the routing decision is based not only on closeness to the target state, but also on the preserved quantum superposition structure. Therefore, REC can be used as an additional reliability indicator that complements E2E fidelity.

Fidelity has traditionally been treated as one of the primary quality indicators for quantum communication links, alongside communication distance^19^. Accordingly, existing routing studies commonly use fidelity as the main reliability criterion for entanglement distribution and routing. More specifically, they rely on link-level or E2E fidelity thresholds to guide path selection, purification planning, and resource allocation, while optimizing additional objectives such as cost efficiency, throughput, resource utilization, distributed operation, or adaptive purification. Although these studies improve fidelity-guaranteed entanglement distribution from different perspectives, they do not explicitly use E2E REC as a reliability parameter and do not evaluate its joint impact on average request success rate (ARSR) and average latency (AL).

Routing studies that mainly examine the joint effects of path selection, entanglement purification, throughput, and resource consumption are predominantly based on the fidelity criterion. Gu et al.^20^ formulate the minimum-cost entanglement distribution and cost-constrained fidelity maximization problems. Their purification and entanglement-swapping graph abstraction jointly represents entanglement generation, purification, the order of entanglement swapping, and their associated costs, thereby enabling the cost-efficient distribution of high-fidelity entanglement. Li et al.^21^ present the purification-enabled Q-PATH and Q-LEAP algorithms for fidelity-guaranteed routing. While Q-PATH searches for paths and purification decisions that provide minimum entangled-pair consumption, Q-LEAP proposes an alternative approach with lower complexity. Zhao et al.^22^ explicitly calculate end-to-end fidelity under a bit-flip error model and present the EFiRAP approach, which jointly determines candidate paths and link-level purification schemes to maximize the number of connections satisfying the required E2E fidelity threshold.

When we look at routing in quantum network studies that accept fidelity as a quality parameter, distributed, memory-free, or computationally efficient approaches are included. Tan et al.^23^ propose DFER, which is a distributed and asynchronous scheme. In this scheme, the E2E fidelity requirement is converted into a link-level fidelity requirement. Moreover, the next hop is selected according to the expected throughput. Yang et al., who propose a multi-tree routing approach based on multiple destination-oriented directed acyclic graphs, extend asynchronous quantum routing in this way^24^. This study improves the E2E entanglement rate in realistic topologies. On the other hand, our proposed approach, CAFARA, focuses on incorporating E2E fidelity and REC into purification-level determination and candidate-path feasibility evaluation. Tunc et al.^3^ developed MEDIRA to achieve fidelity-guaranteed routing in memory-free quantum networks. Tunc et al.^25^ combined centralized and distributed routing approaches within a single routing mechanism. Wang et al.^26^ proposed the SFTRAP approach. According to this approach, fidelity-threshold-aware routing is combined with adaptive purification to improve throughput and reduce routing complexity. Although parameters such as throughput, purification cost, and resource utilization are optimized in the studies mentioned above, their reliability assessments are primarily based on fidelity. These studies do not calculate E2E REC and do not use it as a routing parameter or a request-admission constraint.

In contrast, our proposed CAFARA approach evaluates E2E fidelity and E2E REC together with capacity and latency constraints when determining path feasibility and purification requirements. Therefore, CAFARA extends fidelity-oriented routing by additionally considering the preservation of basis-dependent quantum coherence throughout E2E entanglement distribution.

The main contributions of this paper are summarized as follows:

1. In this study, three different routing strategies developed are compared. In the first algorithm, CAFARA, E2E fidelity and E2E REC are jointly used in purification planning and path selection. In the second algorithm, FARA-PostREC, REC is applied only as a final success condition, while E2E fidelity is used in purification planning. In the third algorithm, FARA-NoREC, the REC constraint is not applied at any stage, and purification planning is performed based on fidelity, just as in FARA-PostREC. The performance of these strategies is evaluated by using ARSR, AL, average purification time (APT), average path length (APL), AFF, and AFC.
2. A physically consistent state-evolution and purification model is developed based on one-sided amplitude damping, Werner-state twirling, and Bennett–Brassard–Popescu–Schumacher–Smolin–Wootters (BBPSSW)^27^ purification. CAFARA selects the minimum feasible purification level that jointly meets the requested E2E fidelity and REC thresholds, subject to the available capacity and latency constraints. On the other hand, the FARA variants determine the purification level based solely on fidelity.

The remainder of this paper is organized as follows. The Results section shows the numerical evaluation of the proposed routing algorithms. This section compares CAFARA with FARA-PostREC and FARA-NoREC for varying channel capacity, inter-node distance, network size, and number of requests. The evaluated metrics are ARSR, AL, APT, APL, AFF, and AFC of successfully served requests. The Discussion section interprets the main findings. Moreover, it examines the trade-offs among request success, fidelity and coherence guarantees, purification overhead, resource consumption, and latency. The Methods section includes the subtitles of one-sided amplitude-damping model, Werner-state twirling, BBPSSW purification, E2E fidelity and REC evaluation, network and request models, feasibility constraints, purification-level determination, path selection, and the overall routing workflow. The Supplementary Information provides detailed notation and performance metric definitions, the complete latency model, the step-by-step workflows for CAFARA, FARA-PostREC, and FARA-NoREC, and their time complexity analyses.

## Results

The software and hardware platform of the simulations in which we evaluated the performance of the routing algorithms we developed for quantum networks is as follows: AMD Ryzen 7 PRO 6850U (8 cores/16 threads) processor, 14 GB of RAM, and Ubuntu Linux (64-bit). All numerical results presented within the scope of the Results section were obtained by repeating each simulation scenario independently 30 times. The confidence interval is reported as 95%. In each run of the scenarios, the range created for the randomly generated request set, link-level probabilistic parameters, and initial fidelity values was implemented using the values given in Table 1.

In our routing algorithms, which account for the dynamic nature of the quantum network by including entanglement swapping, distribution, and purification, we assigned different probabilistic values to various nodes and links to obtain a heterogeneous quantum network. Moreover, we limited the time required for the main steps necessary to process the requests (entanglement generation, purification, and swapping).

In the Waxman model^28^, which we use to generate the connectivity structure of the quantum network, all links are assigned the same physical length in each simulation scenario so that the effect of the inter-node distance can be evaluated independently of each generated graph topology. Therefore, the inter-node distance is a separate, adjustable simulation parameter.

**Table 1:** Simulation parameters, their symbols, and values.

Parameter	Symbol	Value
Coherence threshold	$C_{\textrm{rel}}^{\textrm{th}}$	0.70
Entanglement swapping probability for per intermediate node	ESP	0.95
Number of nodes	*N*	10
Initial fidelity range	$F_{\textrm{inital}}$	*(0.80,\,1)*
Number of requests	$N_{\textrm{req}}$	50
Window time	$T_{\textrm{win}}$	5 s
Traffic demand	$d_x$	10
Fidelity threshold	$F_{\textrm{th}}$	0.80
Memory relaxation time	$T_1$	$1~\textrm{ms}$

In our traffic model, a total of $N_{\textrm{req}}$ connection requests are generated within an arrival window of duration $T_{\textrm{win}}$. Each request *r* is assigned an arrival time $a_r$ drawn independently according to

1 $$\begin{aligned} a_r \sim {\mathscr {U}}(0,T_{\textrm{win}}), \end{aligned}$$

such that all requests arrive within the interval ${[}0,T_{\textrm{win}}]$. For example, the baseline scenario generates $N_{\textrm{req}}=50$ requests within a 5-s arrival window.

The holding time of request *r* is generated as

2 $$\begin{aligned} h_r = \lambda _r T_{\textrm{win}}, \qquad \lambda _r\sim {\mathscr {U}}(0.1,0.9), \end{aligned}$$

and its departure time is defined as

3 $$\begin{aligned} b_r=a_r+h_r. \end{aligned}$$

Therefore, for $T_{\textrm{win}}=5$ s, the holding time lies in the interval $h_r\in [0.5,4.5]$ s and has an expected value of 2.5 s. Each request occupies the allocated network resources during its activity interval $[a_r,b_r]$. Requests whose activity intervals overlap may simultaneously compete for the capacities of shared links in the overlap-aware admission and routing procedure.

Independent of the arrival window, a per-request time bound $T_{\textrm{th}}$ is imposed on the estimated end-to-end processing time of a candidate path, including entanglement generation, purification, and entanglement swapping. A request can be admitted only if the selected path satisfies the required quality-of-service constraints and $T(P)\le T_{\textrm{th}}$. In the present evaluation, $T_{\textrm{th}}=0.005$ s is deliberately selected as a sufficiently loose upper bound. Since the observed end-to-end processing times remain well below this value, the time constraint is non-binding and does not materially affect the reported performance. It is retained as a feasibility safeguard against unrealistically long candidate paths. Therefore, $T_{\textrm{win}}$ primarily controls the temporal distribution and concurrency of requests, whereas $T_{\textrm{th}}$ provides an upper validity bound on per-request processing time.

**Fig. 1:**
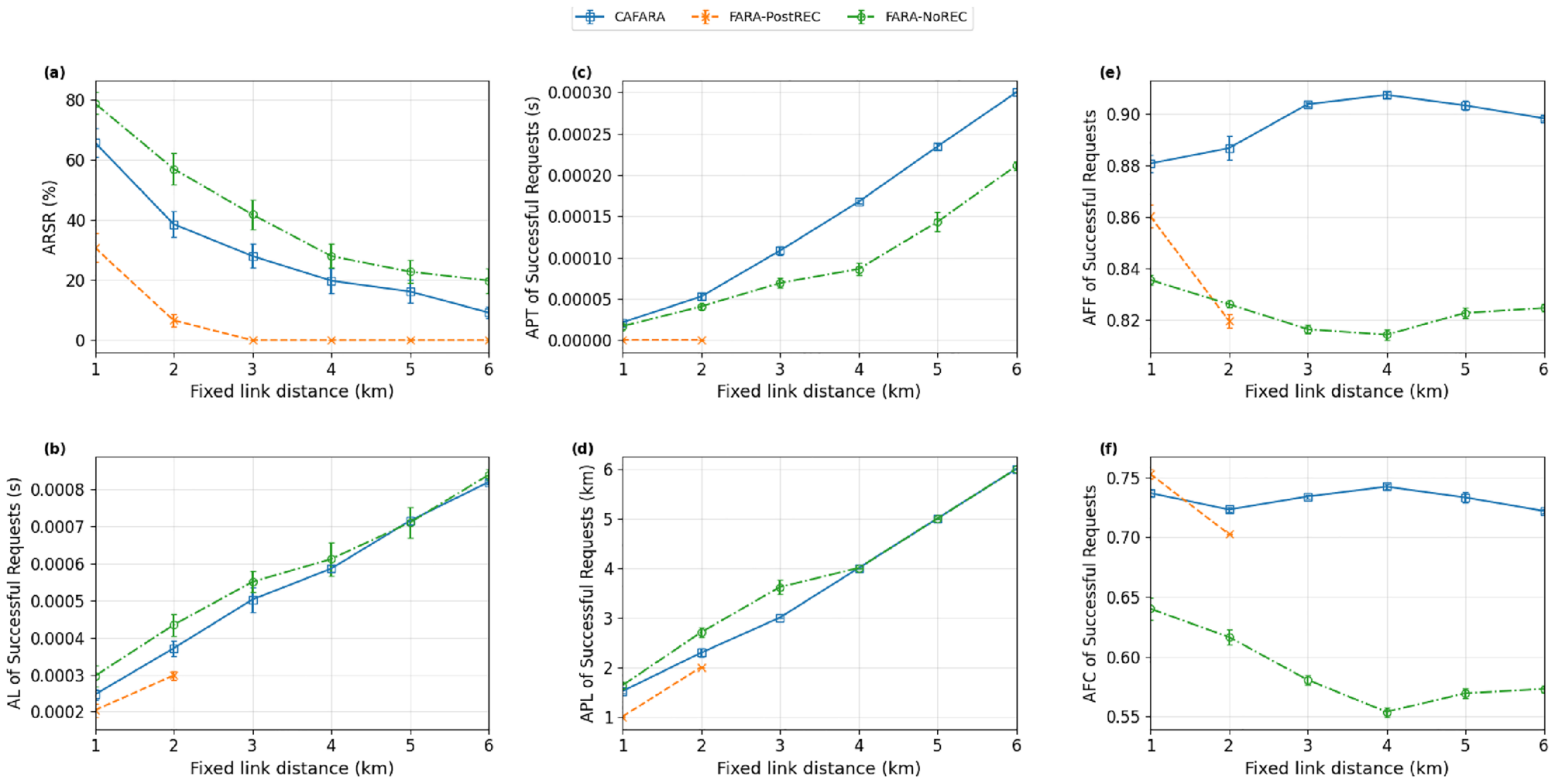
Performance comparison of CAFARA, FARA-PostREC, and FARA-NoREC under varying fixed link distance: (**a**) average request success rate (ARSR), (**b**) average latency of successful requests (AL), (**c**) average purification time of successful requests (APT), (**d**) average path length of successful requests (APL), (**e**) average final fidelity of successful requests (AFF), and (**f**) average final coherence of successful requests (AFC).

1. Performance under Varying Inter-Node Distances:

In Fig. 1, the performance of CAFARA, FARA-PostREC, and FARA-NoREC is compared as the fixed link distance between intermediate nodes varies from 1 km to 6 km. As shown in Fig. 1a, the average request success rate decreases with distance for all three routing strategies. The reason for this decrease is the increase in propagation, entanglement-generation, purification, and swapping times, together with the stronger amplitude-damping effect experienced by the distributed states.

FARA-NoREC achieves the highest request success rate for the investigated distance range. It decreases from approximately *79%* at *1* km to approximately *20%* at *6* km. However, this advantage is expected because FARA-NoREC does not impose an REC constraint during purification planning, path selection, or final request validation. CAFARA exhibits a lower success rate compared to FARA-NoREC. It decreases from approximately *65%* to *9%*, because each accepted request must simultaneously satisfy the fidelity, REC, latency, and capacity constraints.

FARA-PostREC exhibits the lowest success rate. Its success rate decreases from approximately *30%* at *1* km to approximately *6%* at *2* km. Additionally, no successful requests are observed at distances of *3* km or greater. FARA-PostREC determines the purification level without considering REC and checks the REC threshold only after path selection. Consequently, many paths that satisfy the fidelity-based planning conditions are rejected during the final REC validation. In contrast, CAFARA incorporates REC directly into the purification-level determination and can select a higher purification level when necessary, thereby avoiding this late-rejection mechanism.

The average latency of successful requests increases with distance for all strategies, as illustrated in Fig. 1b. CAFARA and FARA-NoREC show similar latency growth, reaching approximately $8.2\times 10^{-4}$ s and $8.4\times 10^{-4}$ s, respectively, at *6* km. The slightly higher latency of FARA-NoREC at some distance values is associated with differences in the paths admitted as successful rather than with a stricter quality-control mechanism.

Figure 1c shows that the average purification time also increases with distance. CAFARA consistently requires more purification time compared to FARA-NoREC because it may continue purification until both the fidelity and REC thresholds are met. At *6* km, the average purification time of CAFARA reaches approximately $3.0\times 10^{-4}$ s, whereas the corresponding value for FARA-NoREC is approximately $2.1\times 10^{-4}$ s. This additional purification effort explains part of the lower request success rate and higher resource consumption of CAFARA. However, CAFARA also produces higher-quality final states.

As shown in Fig. 1d, the average path length of successful requests increases with the fixed link distance. CAFARA and FARA-NoREC exhibit similar path-length behavior over most of the investigated range. This indicates that their differences in final state quality are primarily caused by their purification and REC-handling policies rather than by systematically different path lengths.

The benefits of coherence-aware purification planning are particularly visible in Fig. 1e and f. CAFARA maintains an average final fidelity of approximately *0.88*–*0.91* over the entire distance range, whereas FARA-NoREC remains around *0.81*–*0.84*. Similarly, the average final REC of CAFARA remains relatively stable at approximately *0.72*–*0.74*. At the same time, that of FARA-NoREC decreases from approximately *0.64* at *1* km to about *0.55* at *4* km.

The successful-request fidelity and REC averages of FARA-PostREC are reported only for *1* and *2* km due to no request satisfying all final success conditions beyond *2* km. Therefore, the absence of FARA-PostREC data points at larger distances does not indicate missing simulation results. Rather, the corresponding successful-request averages are undefined because the number of successful requests is zero.

Overall, the distance-sweep results reveal a trade-off among FARA-NoREC, FARA-PostREC, and CAFARA. FARA-NoREC admits more requests because it does not enforce the preservation of coherence. However, the resulting successful states have lower final fidelity and REC. FARA-PostREC shows that applying the REC constraint only after fidelity-based purification and path selection causes severe late-stage rejection. CAFARA reduces the success rate and increases purification time, but it provides significantly stronger and more stable E2E fidelity and REC with increasing link distance.

**Fig. 2:**
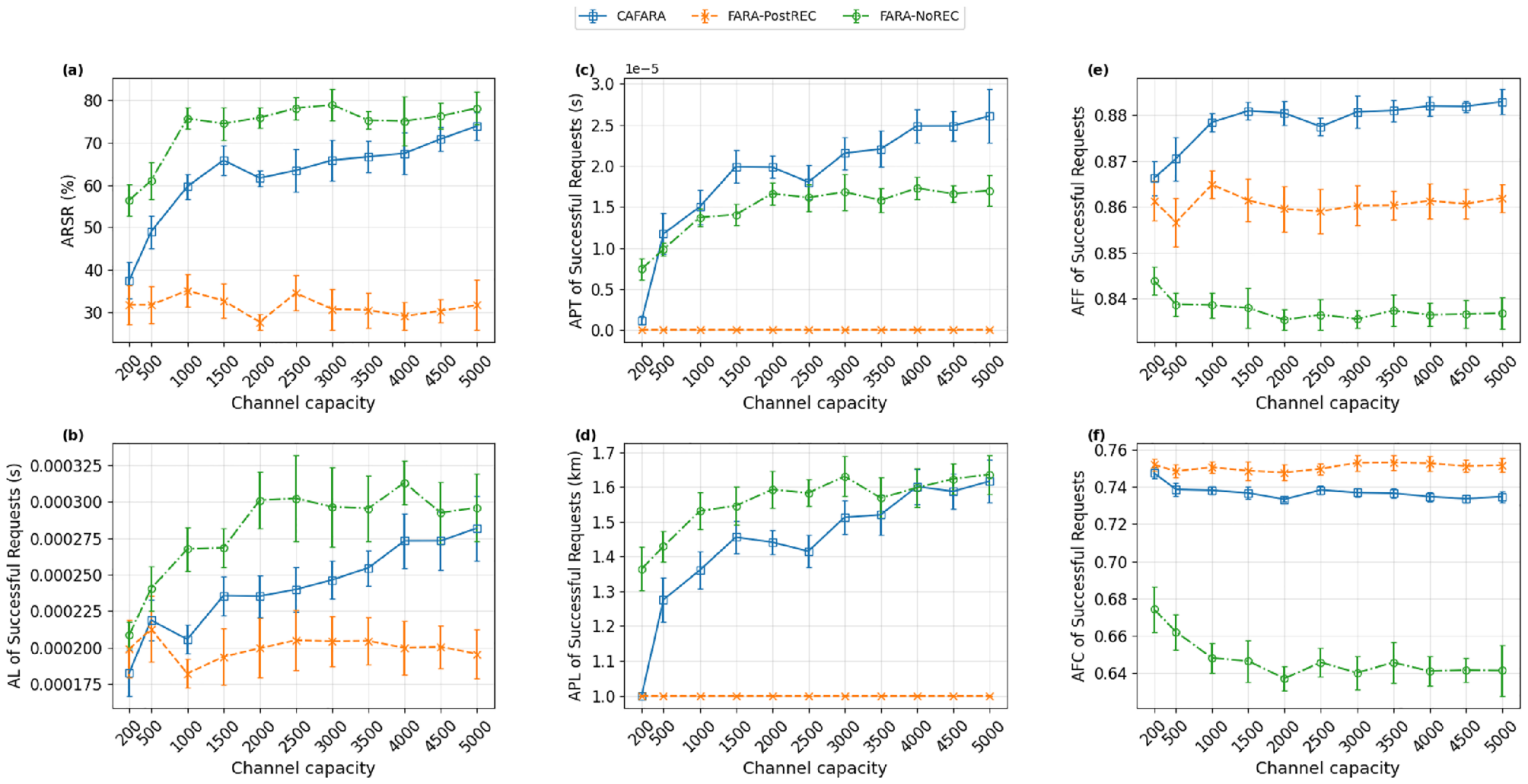
Performance comparison of CAFARA, FARA-PostREC, and FARA-NoREC under varying channel capacity: (**a**) average request success rate (ARSR), (**b**) average latency of successful requests (AL), (**c**) average purification time of successful requests (APT), (**d**) average path length of successful requests (APL), (**e**) average final fidelity of successful requests (AFF), and (**f**) average final coherence of successful requests (AFC).

(2) Performance under Varying Channel Capacity:

Figure 2 compares CAFARA, FARA-PostREC, and FARA-NoREC under channel capacities varying from 200 to 5000 Bell pairs. As shown in Fig. 2a, increasing the available capacity generally improves the ARSR for all three algorithms. FARA-NoREC achieves the highest success rate because it admits requests without enforcing a final coherence requirement. Its success rate increases from approximately *56%* at a capacity of 200 Bell pairs to around *78%* at higher capacities. CAFARA also benefits from the increase in capacity. CAFARA’s ARSR increases from approximately *37%* to *74%*. This improvement occurs because larger capacities allow CAFARA to support purification operations which require more raw Bell pairs, while simultaneously satisfying the fidelity and REC constraints. In contrast, FARA-PostREC remains between approximately *28%* and *35%*. Although additional capacity increases the available resources, FARA-PostREC selects the purification level without considering REC and applies the REC constraint only after path selection. Consequently, many requests are still rejected by the final coherence test even when sufficient capacity is available.

The latency and resource-overhead results are presented in Fig. 2b–d. The average latency of successful requests increases with capacity for CAFARA and FARA-NoREC. This does not imply that larger capacity directly increases the processing time of a request. Rather, higher capacity expands the successful-request set by admitting requests that use longer paths and/or require more purification operations. This interpretation is supported by the increases both in the average purification time and the average path length. For CAFARA, the average path length rises from approximately 1 km to more than 1.6 km, while its purification time also increases as larger-capacity configurations enable more resource-intensive purification levels. FARA-NoREC exhibits a similar, although less pronounced, trend. In contrast, the successful requests under FARA-PostREC have an average path length of approximately 1 km and a purification time close to zero for the considered capacity range. This indicates that the final coherence test predominantly retains direct paths, while rejecting longer paths. This is because their E2E REC does not satisfy the required threshold. This selective admission also explains the comparatively low latency of FARA-PostREC.

Figure 2e and f show the quality of the entangled states of successful requests. CAFARA provides the highest average final fidelity, which remains approximately between 0.87 and 0.88 as capacity increases. FARA-PostREC achieves slightly lower final fidelity, whereas FARA-NoREC produces the lowest fidelity value. In terms of final coherence, FARA-PostREC obtains the highest average values, approximately 0.75. This is because only requests passing the final coherence condition are retained. CAFARA maintains average final coherence values above the required threshold. It admits substantially more requests than FARA-PostREC. FARA-NoREC exhibits the lowest final coherence. This is because REC is neither considered during purification planning nor enforced in the final admission decision.

Overall, the results show that FARA-NoREC maximizes the request admission rate, but the delivered state has low coherence and fidelity. FARA-PostREC provides high coherence and low latency, but its post-selection coherence test results in a substantially lower success rate. CAFARA offers a more balanced operating point due to achieving a considerably higher success rate than FARA-PostREC while maintaining both fidelity and coherence requirements through coherence-aware purification and path selection.

**Fig. 3:**
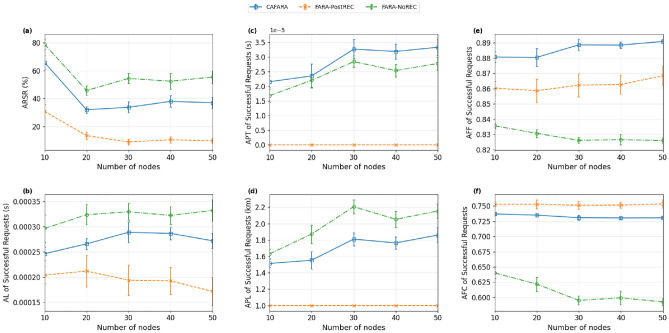
Performance comparison of CAFARA, FARA-PostREC, and FARA-NoREC under varying numbers of quantum nodes: (**a**) average request success rate (ARSR), (**b**) average latency of successful requests (AL), (**c**) average purification time of successful requests (APT), (**d**) average path length of successful requests (APL), (**e**) average final fidelity of successful requests (AFF), and (**f**) average final coherence of successful requests (AFC).

(3) Performance Under Varying the Number of Nodes:

Figure 3 shows the impact of increasing the network size from *10* to *50* quantum nodes when we fixed the capacity at 3000 Bell pairs. For each network size, the topology is regenerated using the same Waxman graph-generation parameters. The number of links is not fixed. Rather, it increases with the number of nodes. However, to prevent larger networks from becoming artificially denser, the networks are generated with a target average node degree of 3 and a minimum node degree of 2.

As shown in Fig. 3a, the average request success rate decreases sharply from *10* to *20* nodes. Then it exhibits smaller fluctuations. Although the number of nodes increases, the target average node degree, link capacity, and link distances are kept fixed. Consequently, the network becomes relatively sparser, and S–D pairs generally require longer paths with more intermediate swapping operations. The resulting increase in path length and latency can be seen in Fig. 3b and d. Moreover, additional swapping operations reduce the cumulative probability of successful swapping. Hence, the usable path capacity is reduced. Moreover, the larger hop count increases decoherence and the difficulty of satisfying the fidelity and REC constraints. Therefore, fewer requests jointly satisfy the quality, capacity, and latency requirements as the network size increases.

FARA-NoREC achieves the highest success rate because it does not impose an REC constraint. However, its average final fidelity and coherence decrease as the network size increases. CAFARA maintains the highest final fidelity and a stable coherence level of approximately *0.73*. However, this level of coherence requires more purification time. As a result, it presents a lower success rate than FARA-NoREC.

FARA-PostREC has the lowest success rate because REC is controlled only after fidelity-based purification and path selection. Its high final coherence, low latency, nearly zero purification time, and short average path length represent the small subset of requests that survive the final REC validation. It doesn’t mean that FARA-PostREC is superior overall in routing performance. The small non-monotonic variations observed beyond *20* nodes arise from differences in the generated network topologies and their available candidate paths.

**Fig. 4:**
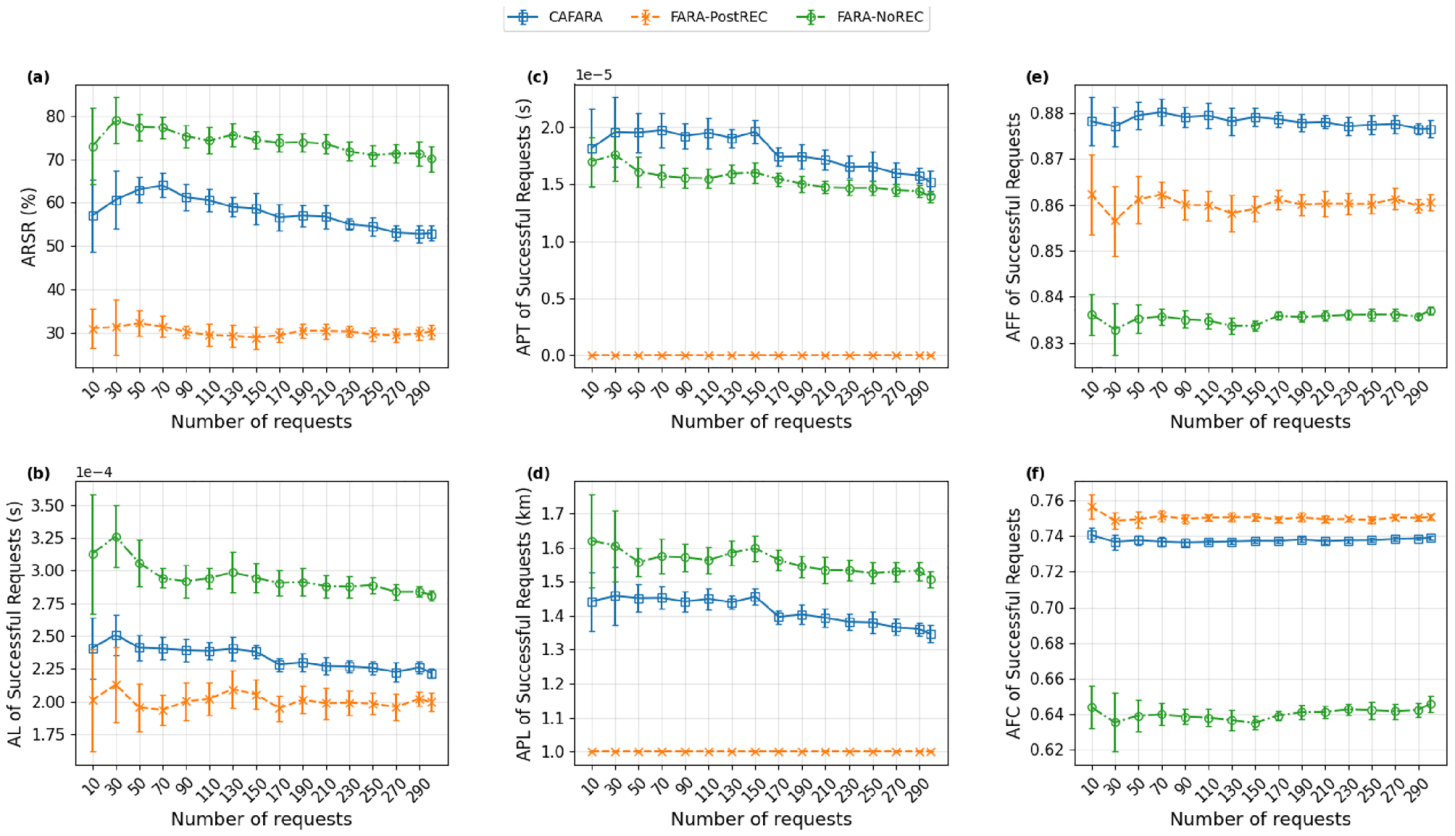
Performance comparison of CAFARA, FARA-PostREC, and FARA-NoREC under varying numbers of connection requests: (**a**) average request success rate (ARSR), (**b**) average latency of successful requests (AL), (**c**) average purification time of successful requests (APT), (**d**) average path length of successful requests (APL), (**e**) average final fidelity of successful requests (AFF), and (**f**) average final coherence of successful requests (AFC).

(4) Performance Under Varying the Number of Requests:

Figure 4 evaluates the three routing strategies when we increased the number of requests. For this scenario, we fixed the network capacity (3000 Bell pairs) and the request-generation window. As the offered load increases, the ARSR of CAFARA and FARA-NoREC generally decreases. This is because of a larger number of temporally overlapping requests competing for the same link capacities. FARA-NoREC achieves the highest ARSR since it does not impose a final REC requirement. On the other hand, FARA-PostREC exhibits the lowest ARSR. Because the final REC constraint for FARA-PostREC may subsequently reject paths selected using fidelity-only planning. CAFARA consistently outperforms FARA-PostREC in terms of ARSR by integrating both fidelity and REC requirements during purification-level determination and candidate-path feasibility evaluation.

The AL, APT, and APL values of successful requests generally decrease when we increase the number of requests. This behavior results from the composition of the successful-request set. Under stronger contention, longer paths and purification configurations requiring greater capacity are more likely to be rejected. This leaves shorter and less resource-intensive requests among the successfully served requests. CAFARA maintains the highest AFF, while its AFC remains above the required REC threshold. Although FARA-PostREC yields a high AFC among its successful requests, this result is accompanied by a substantially lower ARSR due to requests that fail the post-selection REC check being excluded.

**Fig. 5:**
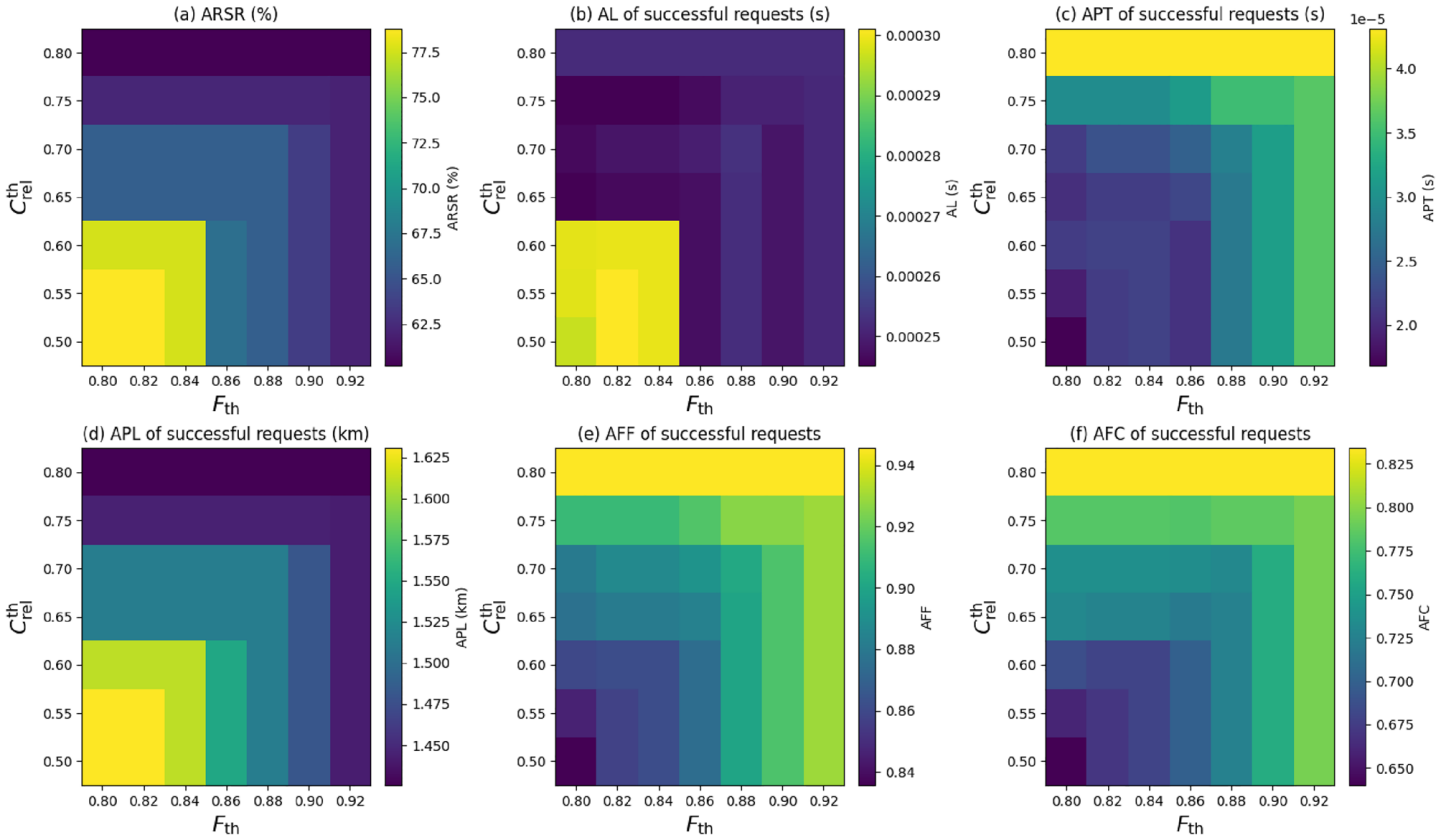
Performance of CAFARA under jointly varying end-to-end fidelity and relative entropy of coherence thresholds: (**a**) average request success rate (ARSR), (**b**) average latency of successful requests (AL), (**c**) average purification time of successful requests (APT), (**d**) average path length of successful requests (APL), (**e**) average final fidelity of successful requests (AFF), and (**f**) average final coherence of successful requests (AFC).

(5) CAFARA Performance Under Joint Fidelity and Coherence Thresholds:

Figure 5 shows the performance of CAFARA when the end-to-end fidelity threshold, $F_{\textrm{th}}$, and the relative entropy of coherence threshold, $C_{\textrm{rel}}^{\textrm{th}}$, are varied concurrently.

As shown in Fig. 5a, the average request success rate decreases when both of the thresholds are increased. This reduction is particularly pronounced with increasing $C_{\textrm{rel}}^{\textrm{th}}$. This indicates that the coherence requirement is often the dominant feasibility constraint. At low coherence thresholds, increasing $F_{\textrm{th}}$ reduces the ARSR. This happens because additional purification operations or higher-quality paths are required in this situation.

Figure 5b shows that the average latency of successful requests is higher when both thresholds are relatively low. And this AL decreases when the thresholds ($F_{\textrm{th}}$ and $C_{\textrm{rel}}^{\textrm{th}}$ ) are increased. This behavior does not show that stricter quality requirements make request processing faster. Rather, low thresholds allow longer and more resource-intensive paths to remain in the successful-request set. On the other hand, stricter thresholds (increased thresholds) reject many such paths. As a result, these stricter thresholds cause a smaller subset that is dominated by shorter and more time-efficient connections.

The purification-time results in Fig. 5c show a different trend. The average purification time increases substantially with $C_{\textrm{rel}}^{\textrm{th}}$. On the other hand, the effect of $F_{\textrm{th}}$ is comparatively weaker. A higher coherence requirement can force CAFARA to continue purification even after the fidelity constraint has been satisfied. Conversely, increasing $F_{\textrm{th}}$ mainly affects purification time when the coherence threshold is low or moderate. The block-like structure on top of the figure results from the discrete nature of the selected BBPSSW purification level.

Figure 5d confirms the successful-request selection effect observed in the latency results. The average path length is largest when both thresholds are low. This happens because longer multi-hop paths can still satisfy the relaxed quality requirements. When the thresholds increase, longer paths are rejected more. This is because they experience greater decoherence, more swapping operations, and higher raw Bell-pair requirements. Consequently, the successful-request set becomes increasingly dominated by shorter paths. This reduction in path length partly compensates for the higher purification overhead and explains why the total latency may decrease even when the purification time increases.

The quality of the successfully delivered entangled states is shown in Fig. 5e and f. Both the average final fidelity and the average final coherence increase as the thresholds increase. Increasing $F_{\textrm{th}}$ directly removes low-fidelity solutions and may require additional purification. On the other hand, increasing $C_{\textrm{rel}}^{\textrm{th}}$ similarly eliminates low-coherence paths and often leads to higher purification levels. At high coherence thresholds, the influence of $F_{\textrm{th}}$ becomes less pronounced because the coherence condition already enforces the selection of highly purified states having both high fidelity and high REC.

Overall, the joint-threshold results reveal a clear trade-off. Increased fidelity and coherence requirements improve the final state quality but reduce the number of successfully served requests. Moreover, they increase the purification overhead. At the same time, stricter thresholds preferentially retain shorter paths, which lowers the average path length and the conditional latency of the successful-request set. The stronger horizontal band structure observed across several panels further indicates that CAFARA is generally more sensitive to $C_{\textrm{rel}}^{\textrm{th}}$ than to $F_{\textrm{th}}$. Therefore, the two quality thresholds should be configured jointly to balance request admission, purification cost, latency, and the quality of the delivered entangled states.

## Discussion

This study shows that incorporating the E2E REC into purification planning and routing decisions provides a more comprehensive quality-control mechanism compared to relying on fidelity alone. CAFARA evaluates the E2E fidelity and REC associated with every candidate purification level, and it selects the minimum level of purification that jointly satisfies the fidelity, coherence, latency, and capacity constraints. Our baseline routing algorithm, FARA-PostREC, determines the purification level and selects the path without using REC. It applies the REC requirement only during final request validation. FARA-NoREC does not use REC during purification planning, path selection, or final request evaluation.

The results reveal a fundamental trade-off between request admission and the quality guarantees provided for the delivered entangled states. Among the evaluated scenarios, FARA-NoREC generally achieves the highest ARSR due to applying the least restrictive success criterion. However, this admission advantage is obtained without guaranteeing the final E2E REC threshold. Its successful requests consistently exhibit the lowest average final coherence and, in most cases, the lowest average final fidelity. Its ARSR should therefore be interpreted as the success rate of a fidelity-only strategy rather than as a quality-equivalent improvement over CAFARA.

In the FARA-PostREC algorithm, REC is used only as a final selection constraint. The REC value is not checked for the candidate paths found initially. Therefore, even if the selected candidate path satisfies the fidelity, capacity, and latency thresholds, it can be rejected if it does not satisfy the REC threshold in the final evaluation. If there is no other candidate path after this rejection, the request is dropped. This dropping process is particularly evident for longer paths, i.e., multi-hop paths. As a result, FARA-PostREC provides the lowest ARSR for the investigated scenarios. On the other hand, CAFARA includes REC in the determination process of the purification operation and reduces request drops that may occur because of the REC threshold at the final stage. Based on these reasons, CAFARA consistently provides a higher ARSR than FARA-PostREC while satisfying the same final fidelity and REC requirements.

At this point, the relatively high AFC we reported for FARA-PostREC should be considered together with the low ARSR provided by FARA-PostREC. Since requests with insufficient E2E REC are eliminated at the final selection stage, only a small subset with high REC is considered successful in the obtained successful-request average. Therefore, the high AFC of FARA-PostREC is not evidence that the fidelity-only routing procedure preserves coherence more effectively. On the contrary, this situation is mostly a sign that direct paths requiring no swapping are selected, that is, surviving paths are selected, and the purification process is avoided. On the other hand, CAFARA successfully delivers a significantly larger proportion of requests to the destination compared with FARA-PostREC while keeping its average final coherence above the required REC threshold.

APT, APL, and ARSR obtained from the data in this study should be interpreted together. The elements that constitute the total E2E latency are entanglement generation time, entanglement purification time, and entanglement swapping time. Therefore, a larger APT does not mean that the total AL increases by the same amount. In some scenarios, CAFARA selects shorter or more time-efficient paths than FARA-NoREC. Thus, the corresponding reductions in entanglement generation and swapping times can partially compensate for the additional purification overhead occurring in CAFARA to satisfy the REC threshold. Nevertheless, CAFARA does not provide the lowest latency in every experiment. It should be stated that FARA-PostREC frequently exhibits the lowest AL, an APT close to zero, and an APL close to one link. The reason for this obtained result is that the successful request set of FARA-PostREC consists of short and direct paths requiring little or no purification. As a result, the low latency of this small surviving subset is obtained thanks to a lower request success rate.

When the results obtained by increasing the capacity are evaluated, it is clear that the additional channel capacity has a positive effect on the ARSR value of the routing algorithms. For CAFARA, more capacity means enabling the purification process that requires more raw Bell pairs. As a result of this process, longer paths that jointly satisfy the E2E fidelity and REC requirements can be added to the successful request set. At the same time, since a larger capacity allows more purification and longer paths, it can also increase the AL, APT, and APL values measured for successful requests. Therefore, these increases should be understood as a change in the composition of the successful request set rather than as a direct increase in the processing time caused by the capacity itself.

In the CAFARA approach, the minimum purification level satisfying all applicable constraints is selected. As a result, the number of raw Bell pairs used is optimized. However, although the fidelity requirement is satisfied, when the REC requirement is not satisfied, CAFARA may select a higher purification level than the FARA algorithms that select the purification level based only on fidelity. Thus, the expected raw-pair consumption may increase, and the capacity available for concurrent requests may decrease. This situation explains the trade-off between stronger E2E quality guarantees and request admission. In resource-constrained environments, adjustments can be made with appropriate REC and fidelity thresholds in an adaptive policy, and these thresholds can be increased when resources are abundant.

The routing algorithms we developed (CAFARA, FARA-PostREC, and FARA-NoREC) are based on a centralized, on-demand routing model, equal link distances within each simulation scenario, one-sided amplitude damping, Werner-state twirling, and BBPSSW purification. The physical-layer model is a routing-level abstraction that includes imperfect initial states, amplitude damping, probabilistic BBPSSW purification, and swapping success probabilities. However, local gate and measurement errors, detector imperfections, and additional memory decoherence caused by asynchronous waiting are not explicitly modeled. Additionally, swapping failures are represented through expected success probabilities rather than event-by-event simulation.

Quantum routing architectures managed by a central controller are vulnerable to single points of failure and compromised repeaters^29^. Therefore, future studies will address distributed and/or multipath routing, heterogeneous link distances, additional physical noise and purification models, adaptive fidelity and coherence thresholds, and multi-objective path selection. In particular, asynchronous topology-maintenance mechanisms, such as the distributed Destination-Oriented Directed Acyclic Graph (DODAG)- and spanning-tree-based approaches proposed in^30^, may enable coherence- and fidelity-aware decisions to be extended beyond the centralized on-demand model considered here.

As a result, the main aim of CAFARA is to increase the number of transmitted requests by keeping the quality of the transmitted state above a certain threshold. To achieve this aim, CAFARA provides a proactive, quality-aware operating point by jointly considering fidelity, REC, latency, and capacity before path selection. When we examine the simulation results we obtained, we see that, thanks to this approach, the late coherence-related rejections observed in FARA-PostREC are significantly reduced in CAFARA. Moreover, the required E2E fidelity and coherence are preserved, and a more balanced trade-off among quality-guaranteed request success, purification overhead, resource consumption, path length, and E2E latency is achieved.

## Methods

In this section, we present the physical-state and routing models used in this study. We first describe the calculation of E2E REC under imperfect initial states, one-sided amplitude damping, twirling, BBPSSW purification, and entanglement swapping. We then introduce the network and request models, path-feasibility and resource constraints, purification planning for CAFARA and FARA, the complete routing workflow, and the entanglement generation rate used for path selection. Detailed algorithmic procedures and time-complexity analyses for CAFARA, FARA-PostREC, and FARA-NoREC are included in the Supplementary Information.

### Calculation of E2E REC

In this study, quantum coherence is quantified using REC, as introduced by Baumgratz et al.^31^. Since REC is basis-dependent, all coherence values are evaluated with respect to the fixed two-qubit computational basis

4 $$\begin{aligned} {\mathscr {B}} = \left\{ |00\rangle , |01\rangle , |10\rangle , |11\rangle \right\} . \end{aligned}$$

For a two-qubit density matrix *ρ*, REC is defined as^31^

5 $$\begin{aligned} C_{\textrm{rel}}(\rho ) = S\left( \rho _{\textrm{diag}}\right) - S(\rho ), \end{aligned}$$

where

6 $$\begin{aligned} S(\rho ) = -\operatorname {Tr} \left[ \rho \log _{2}(\rho ) \right] \end{aligned}$$

is the von Neumann entropy, and

7 $$\begin{aligned} \rho _{\textrm{diag}} = \sum _i \langle i|\rho |i\rangle |i\rangle \langle i| \end{aligned}$$

is the completely dephased state in the computational basis. The logarithm is taken in base two; hence, REC is expressed in bits.

The maximally entangled Bell state used as the target state for fidelity evaluation is

8 $$\begin{aligned} |\Phi ^{+}\rangle = \frac{|00\rangle +|11\rangle }{\sqrt{2}}, \qquad \rho _{\Phi ^{+}} = |\Phi ^{+}\rangle \langle \Phi ^{+}|. \end{aligned}$$

With respect to the basis in Eq. (4), $C_{\textrm{rel}}(\rho _{\Phi ^{+}})=1$. However, the elementary entangled pairs generated over the network links are not assumed to be perfect Bell states.

*Imperfect initial entangled state.* The initial state of an elementary entangled pair is represented by a Werner state with initial fidelity $F_{\textrm{initial}}$ with respect to $|\Phi ^{+}\rangle$^27,32^:

9 $$\begin{aligned} \begin{aligned} \rho _{\textrm{initial}} \left( F_{\textrm{initial}} \right) ={}&F_{\textrm{initial}} |\Phi ^{+}\rangle \langle \Phi ^{+}|\\&+ \frac{1-F_{\textrm{initial}}}{3} \Big ( |\Phi ^{-}\rangle \langle \Phi ^{-}|+ |\Psi ^{+}\rangle \langle \Psi ^{+}|+ |\Psi ^{-}\rangle \langle \Psi ^{-}|\Big ). \end{aligned} \end{aligned}$$

The remaining Bell states are

10 $$\begin{aligned} \begin{aligned} |\Phi ^{-}\rangle&= \frac{|00\rangle -|11\rangle }{\sqrt{2}}, \\ |\Psi ^{+}\rangle&= \frac{|01\rangle +|10\rangle }{\sqrt{2}}, \\ |\Psi ^{-}\rangle&= \frac{|01\rangle -|10\rangle }{\sqrt{2}}. \end{aligned} \end{aligned}$$

In the numerical evaluation, the initial elementary-link fidelity is varied within the interval

11 $$\begin{aligned} F_{\textrm{initial}} \in [0.8,1.0]. \end{aligned}$$

The elementary links of a candidate path have different assigned initial fidelities, the lookup-based path evaluation uses the conservative effective initial fidelity

12 $$\begin{aligned} F_{\textrm{initial}}^{{\mathscr {P}}} = \min _{\ell \in {\mathscr {P}}} F_{\textrm{initial}}^{(\ell )}, \end{aligned}$$

where $F_{\textrm{initial}}^{(\ell )}$ is the initial fidelity of elementary link *ℓ*. This ensures that the path-level prediction does not overestimate the quality of the weakest elementary link.

For the online lookup-based evaluation, the heterogeneous link parameters are represented by effective path-level quantities. In addition to the weakest-link initial fidelity in Eq. (12), the amplitude-damping exposure of candidate path *P* is represented by $\tau ^{P}$ and is defined from the path-level entanglement-generation time $T_{\textrm{ent}}(P)$. The parameter $T_1$ denotes the characteristic energy-relaxation time of the amplitude-damping channel. Therefore, $\tau ^{P}/T_1$ represents the normalized damping exposure used in the lookup-based state evaluation:

13 $$\begin{aligned} \tau ^{P} = T_{\textrm{ent}}(P), \qquad \frac{\tau ^{P}}{T_1} = \frac{T_{\textrm{ent}}(P)}{T_1}. \end{aligned}$$

Thus, each online lookup query uses the conservative path-level initial fidelity $F_{\textrm{initial}}^{P}$ together with the normalized path-level exposure duration $\tau ^{P}/T_1$.

*One-sided amplitude-damping model.*  The decoherence of an elementary entangled pair before purification is modeled using the amplitude-damping channel, which represents irreversible energy relaxation. Amplitude-damping Kraus operators are also used in density-matrix evaluations of entanglement purification under realistic noise conditions^33,34^:

14 $$\begin{aligned} E_{0}(\gamma ) = \begin{pmatrix} 1 & 0\\ 0 & \sqrt{1-\gamma } \end{pmatrix}, \qquad E_{1}(\gamma ) = \begin{pmatrix} 0 & \sqrt{\gamma }\\ 0 & 0 \end{pmatrix}, \end{aligned}$$

where $\gamma \in [0,1]$ is the amplitude-damping probability. Under the standard Markovian amplitude-damping model, its dependence on the exposure time *τ* is given by^33,34^

15 $$\begin{aligned} \gamma (\tau ) = 1-\exp \left( -\frac{\tau }{T_{1}}\right) , \end{aligned}$$

where $T_{1}$ is the characteristic energy-relaxation time. In the simulations, the characteristic energy-relaxation time is set to $T_1=10^{-3}\,\textrm{s}$, i.e., $1\,\textrm{ms}$. This parameter defines the time scale of the amplitude-damping process, where the damping probability depends on the normalized storage time $\tau /T_1$.

We adopt a one-sided amplitude-damping model in which an imperfect entangled pair is generated locally, qubit *A* remains at the source, and qubit *B* is transmitted through the noisy quantum channel. The model is based on the standard Kraus operator-sum representation of a quantum operation^33^. Let *σ* denote an arbitrary single-qubit input density matrix. For a single-qubit amplitude-damping channel, the output state can be written as

16 $$\begin{aligned} {\mathscr {E}}_{\textrm{AD}}(\sigma ) = \sum _{\nu =0}^{1} E_\nu (\gamma )\,\sigma \,E_\nu ^\dagger (\gamma ) = E_0(\gamma )\sigma E_0^\dagger (\gamma ) + E_1(\gamma )\sigma E_1^\dagger (\gamma ). \end{aligned}$$

where $E_\nu (\gamma )$, with $\nu \in \{0,1\}$, are the Kraus operation elements of the amplitude-damping channel, and *γ* is the corresponding amplitude-damping probability^33^. In the present two-qubit setting, this channel is applied only to the transmitted qubit *B*, while qubit *A* evolves trivially. Therefore, the corresponding two-qubit Kraus operators are

17 $$\begin{aligned} K_\nu (\tau )=I_A \otimes E_\nu (\gamma (\tau )), \qquad \nu \in \{0,1\}. \end{aligned}$$

where $I_A$ denotes the identity operator acting on qubit *A*, and $E_\nu (\gamma (\tau ))$ is the amplitude-damping Kraus operator acting on the transmitted qubit *B*.

The resulting time-dependent elementary-link state is then given by

18 $$\begin{aligned} \begin{aligned} \rho _{\textrm{AD}} \left( \tau ; F_{\textrm{initial}} \right) = \sum _{\nu =0}^{1} K_\nu (\tau ) \rho _{\textrm{initial}} \left( F_{\textrm{initial}} \right) K_\nu ^\dagger (\tau )= \sum _{\nu =0}^{1} \left[ I_{A}\otimes E_{\nu }\left( \gamma (\tau )\right) \right] \rho _{\textrm{initial}} \left( F_{\textrm{initial}} \right) \left[ I_{A}\otimes E_{\nu }\left( \gamma (\tau )\right) \right] ^{\dagger }. \end{aligned} \end{aligned}$$

In Eq. 18, $\rho _{\textrm{AD}}(\tau ; F_{\textrm{initial}})$ denotes the density matrix obtained after amplitude damping has acted on the transmitted half of the initially imperfect entangled pair. The operator $I_A$ is the identity operator on qubit *A*, which remains locally at the source node and is not affected by the channel-induced energy-relaxation process. The operators $E_\nu (\gamma (\tau ))$, with $\nu \in {0,1}$, are the amplitude-damping Kraus operators acting on qubit *B*. The two terms in the summation correspond to the two amplitude-damping branches: the no-relaxation branch and the relaxation branch. The damping probability $\gamma (\tau )$ depends on the exposure time *τ* and is governed by the characteristic energy-relaxation time $T_1$. Thus, Eq. 18 implements a one-sided amplitude-damping channel, where only the transmitted qubit of the elementary entangled pair is decohered.

The corresponding elementary-link fidelity and REC are computed from the same amplitude-damped density matrix. The fidelity is evaluated as the overlap with the target Bell state $|\Phi ^{+}\rangle$, following the standard Bell-state fidelity convention used in entanglement purification^27,35^. The REC is obtained by applying the REC definition of Baumgratz et al.^31^ to the amplitude-damped state:

19 $$\begin{aligned} F_{\textrm{AD}} \left( \tau ; F_{\textrm{initial}} \right)= & \langle \Phi ^{+}|\rho _{\textrm{AD}} \left( \tau ; F_{\textrm{initial}} \right) |\Phi ^{+}\rangle , \end{aligned}$$

20 $$\begin{aligned} C_{\textrm{rel}}^{\textrm{AD}}(\tau ;F_{\textrm{initial}})= & S\!\left[ \bigl (\rho _{\textrm{AD}}(\tau ;F_{\textrm{initial}})\bigr )_{\textrm{diag}}\right] - S\!\left[ \rho _{\textrm{AD}}(\tau ;F_{\textrm{initial}})\right] . \end{aligned}$$

*Twirling and BBPSSW purification.* Although the initially generated pair is represented as a Werner state, one-sided amplitude damping does not generally preserve the Werner form. Therefore, before applying the BBPSSW purification, the amplitude-damped state is transformed by random bilateral rotations, commonly referred to as twirling, into a Werner state having the same fidelity with respect to $|\Phi ^{+}\rangle$^27,35^.

The resulting state is

21 $$\begin{aligned} \begin{aligned} \rho _{\textrm{W}} \left( F_{\textrm{AD}} \left( \tau ; F_{\textrm{initial}} \right) \right) ={}&F_{\textrm{AD}} \left( \tau ; F_{\textrm{initial}} \right) |\Phi ^{+}\rangle \langle \Phi ^{+}|\\&+ \frac{ 1- F_{\textrm{AD}} \left( \tau ; F_{\textrm{initial}} \right) }{3} \Big ( |\Phi ^{-}\rangle \langle \Phi ^{-}|+ |\Psi ^{+}\rangle \langle \Psi ^{+}|+ |\Psi ^{-}\rangle \langle \Psi ^{-}|\Big ). \end{aligned} \end{aligned}$$

Twirling preserves fidelity but does not generally preserve REC. Therefore, the coherence of the BBPSSW input state is recomputed as

22 $$\begin{aligned} C_{\textrm{rel}}^{\textrm{W}} = S\!\left[ \left( \rho _{\textrm{W}}\!\left( F_{\textrm{AD}}(\tau ;F_{\textrm{initial}})\right) \right) _{\textrm{diag}}\right] - S\!\left[ \rho _{\textrm{W}}\!\left( F_{\textrm{AD}}(\tau ;F_{\textrm{initial}})\right) \right] . \end{aligned}$$

When no purification is applied, coherence is evaluated directly from $\rho _{\textrm{AD}} (\tau ;F_{\textrm{initial}})$, and no twirling operation is included in the final link state. When purification is applied, two identical Werner states are used as the source and target pairs of one BBPSSW round. Both parties apply local controlled-NOT operations from the source pair to the target pair and subsequently measure the target qubits in the computational basis^27,35^. In one BBPSSW purification round, two noisy elementary pairs are used. We denote the pair to be retained, conditioned on a successful purification outcome, by $(A_1,B_1)$, and the auxiliary target pair to be measured by $(A_2,B_2)$. Alice applies a local CNOT from $A_1$ to $A_2$, while Bob applies a local CNOT from $B_1$ to $B_2$. The target qubits $A_2$ and $B_2$ are then measured in the computational basis, producing the classical outcomes $m_{A_2}$ and $m_{B_2}$, respectively. The retained pair $(A_1,B_1)$ is kept only when these outcomes coincide:

23 $$\begin{aligned} m_{A_{2}} = m_{B_{2}}, \qquad m_{A_{2}},m_{B_{2}}\in \{0,1\}. \end{aligned}$$

The BBPSSW recurrence is initialized with the fidelity of the amplitude-damped elementary-link state:

24 $$\begin{aligned} F_{0} = F_{\textrm{AD}} \left( \tau ; F_{\textrm{initial}} \right) . \end{aligned}$$

Here, $F_0$ represents the pre-purification fidelity after amplitude damping, whereas $F_k$ for *k\ge 1* denotes the Werner-state fidelity after *k* successful BBPSSW purification rounds.

For two identical Werner-state inputs, the BBPSSW success probability is^27,35^

25 $$\begin{aligned} P_{\textrm{BBPSSW}}(F_{k}) = F_{k}^{2} + \frac{2}{3}F_{k}(1-F_{k}) + \frac{5}{9}(1-F_{k})^{2}. \end{aligned}$$

Conditioned on success, the fidelity is updated according to the standard BBPSSW recurrence relation^27,35^:

26 $$\begin{aligned} F_{k+1} = \frac{ F_{k}^{2} + \frac{1}{9}(1-F_{k})^{2} }{ F_{k}^{2} + \frac{2}{3}F_{k}(1-F_{k}) + \frac{5}{9}(1-F_{k})^{2} }. \end{aligned}$$

For $F_{k}>1/2$, the BBPSSW recurrence increases the Werner-state fidelity. After each successful round, the retained pair is again represented in Werner form:

27 $$\begin{aligned} \rho _{\textrm{W}}^{(k+1)} = \rho _{\textrm{W}}(F_{k+1}), \end{aligned}$$

and its REC is recomputed as

28 $$\begin{aligned} C_{\textrm{rel}}^{(k+1)} = S\left[ \left( \rho _{\textrm{W}}^{(k+1)} \right) _{\textrm{diag}} \right] - S\left[ \rho _{\textrm{W}}^{(k+1)} \right] . \end{aligned}$$

For *M* successful purification rounds, Eq. (26) is applied iteratively for *k=0,… ,M-1*, yielding the link-level quantities $F_{M}$ and $C_{\textrm{rel}}^{(M)}$.

*End-to-end state construction.*  Let ${\mathscr {P}}=(v_{0},v_{1},\ldots ,v_{X})$ denote a path containing *X* elementary links and *X-1* intermediate nodes. For the lookup-based path evaluation, the state of elementary link *ℓ* is represented using the effective path fidelity $F_{\textrm{initial}}^{{\mathscr {P}}}$ as

29 $$\begin{aligned} \rho _{\ell }^{(M)} \left( \tau ^P; F_{\textrm{initial}}^{{\mathscr {P}}} \right) = {\left\{ \begin{array}{ll} \rho _{\textrm{AD}} \left( \tau ^{P}; F_{\textrm{initial}}^{{\mathscr {P}}} \right) , & M=0, \\ \rho _{\textrm{W}} \left( F_{M} \left( \tau ^{P}; F_{\textrm{initial}}^{{\mathscr {P}}} \right) \right) , & M\ge 1, \end{array}\right. } \qquad \ell =1,\ldots ,X. \end{aligned}$$

The index *ℓ* indicates only the position of the effective elementary state along the path. All effective elementary links used in the lookup-based state construction are evaluated using the same path-level parameters $F_{\textrm{initial}}^{{\mathscr {P}}}$ and $\tau ^{P}/T_1$.

Consider two adjacent states $\rho _{AB_{1}}$ and $\rho _{B_{2}C}$, where $B_{1}$ and $B_{2}$ belong to the intermediate node. Entanglement swapping is performed by a Bell-state measurement on these two qubits^35,36^. Before measurement, the joint state is

30 $$\begin{aligned} \rho _{AB_{1}B_{2}C} = \rho _{AB_{1}} \otimes \rho _{B_{2}C}. \end{aligned}$$

For a Bell-state outcome $\beta \in \{\Phi ^{+},\Phi ^{-},\Psi ^{+},\Psi ^{-}\}$, the measurement projector is

31 $$\begin{aligned} \Pi _{\beta } = I_{A} \otimes |\beta \rangle \langle \beta |_{B_{1}B_{2}} \otimes I_{C}. \end{aligned}$$

In Eq. 31, $\Pi _{\beta }$ is the measurement projector corresponding to the Bell-state outcome $\beta \in \{\Phi ^{+},\Phi ^{-},\Psi ^{+},\Psi ^{-}\}$. The operators $I_A$ and $I_C$ denote identity operators on the remote qubits *A* and *C*, respectively, while $|\beta \rangle \langle \beta |_{B_1B_2}$ acts on the two qubits $B_1$ and $B_2$ measured at the intermediate node. Thus, the projector acts nontrivially only on the measured qubits and leaves the end qubits unchanged.

The corresponding unnormalized remote state and outcome probability are obtained using the standard projective-measurement rule^33,36^:

32 $$\begin{aligned} {\widetilde{\rho }}_{AC}^{(\beta )}= & \operatorname {Tr}_{B_{1}B_{2}} \left[ \Pi _{\beta } \rho _{AB_{1}B_{2}C} \Pi _{\beta } \right] , \end{aligned}$$

33 $$\begin{aligned} p_{\beta }= & \operatorname {Tr} \left[ {\widetilde{\rho }}_{AC}^{(\beta )} \right] . \end{aligned}$$

After normalization and the outcome-dependent Pauli correction $U_{\beta }$ on qubit *C*, the corrected remote state is^33,35^

34 $$\begin{aligned} \rho _{AC}^{(\beta )} = \left( I_{A}\otimes U_{\beta } \right) \frac{ {\widetilde{\rho }}_{AC}^{(\beta )} }{ p_{\beta } } \left( I_{A}\otimes U_{\beta } \right) ^{\dagger }. \end{aligned}$$

Let ${\mathscr {B}}_{\textrm{id}}$ denote the set of Bell-state measurement outcomes that are successfully identified by the swapping operation. Following the standard post-measurement state-update rule for projective measurements^33^ and the Bell-state-measurement-based entanglement-swapping model^35,36^, the total probability of obtaining a successfully identified Bell outcome is

35 $$\begin{aligned} P_{\textrm{BSM}}^{\textrm{id}} = \sum _{\beta \in {\mathscr {B}}_{\textrm{id}}} p_{\beta }. \end{aligned}$$

Accordingly, the state conditioned on a successful Bell-state measurement is the normalized mixture of the Pauli-corrected remote states associated with the successfully identified Bell outcomes:

36 $$\begin{aligned} \operatorname {Swap} \left( \rho _{AB_{1}}, \rho _{B_{2}C} \right) = \frac{ \displaystyle \sum _{\beta \in {\mathscr {B}}_{\textrm{id}}} p_{\beta }\rho _{AC}^{(\beta )} }{ \displaystyle P_{\textrm{BSM}}^{\textrm{id}} } = \frac{ \displaystyle \sum _{\beta \in {\mathscr {B}}_{\textrm{id}}} p_{\beta }\rho _{AC}^{(\beta )} }{ \displaystyle \sum _{\beta \in {\mathscr {B}}_{\textrm{id}}} p_{\beta } }. \end{aligned}$$

Here $P_{\textrm{BSM}}^{\textrm{id}}$ is the total probability of the successfully identified Bell-measurement branches and acts as the normalization factor of the conditional swapped state , $p_{\beta }$ is the probability of Bell-state outcome *β*, and $\rho _{AC}^{(\beta )}$ is the corresponding normalized and Pauli-corrected remote state. The denominator in Eq. 36 normalizes the conditional state over the successfully identified Bell-measurement branches. The network-level entanglement-swapping success probability is treated separately in the routing and resource-admission model.

The first state in the recursive path-level swapping procedure is

37 $$\begin{aligned} \rho _{v_{0}v_{1}}^{(M,1)} = \rho _{1}^{(M)} \left( \tau ^{P}; F_{\textrm{initial}}^{{\mathscr {P}}} \right) . \end{aligned}$$

For *j=1,… ,X-1*, the current long-distance state is connected to the next elementary link according to

38 $$\begin{aligned} \rho _{v_{0}v_{j+1}}^{(M,j+1)} = \operatorname {Swap}\!\left( \rho _{v_{0}v_{j}}^{(M,j)}, \rho _{j+1}^{(M)}\!\left( \tau ^{P};F_{\textrm{initial}}^{{\mathscr {P}}}\right) \right) . \end{aligned}$$

After all *X-1* swapping operations, the end-to-end state is

39 $$\begin{aligned} \rho _{\textrm{E2E}}^{(M,X)} \left( F_{\textrm{initial}}^{{\mathscr {P}}} \right) = \rho _{v_{0}v_{X}}^{(M,X)}. \end{aligned}$$

For *X=1*, no swapping is required and

40 $$\begin{aligned} \rho _{\textrm{E2E}}^{(M,1)} = \rho _{1}^{(M)} \left( \tau ^{P}; F_{\textrm{initial}}^{{\mathscr {P}}} \right) . \end{aligned}$$

Finally, end-to-end fidelity and REC are computed from the same final density matrix:

41 $$\begin{aligned} F_{\textrm{E2E}}^{(M,X)}= & \langle \Phi ^{+}|\rho _{\textrm{E2E}}^{(M,X)} |\Phi ^{+}\rangle , \end{aligned}$$

42 $$\begin{aligned} C_{\textrm{rel,E2E}}^{(M,X)}= & S\left[ \left( \rho _{\textrm{E2E}}^{(M,X)} \right) _{\textrm{diag}} \right] - S\left[ \rho _{\textrm{E2E}}^{(M,X)} \right] . \end{aligned}$$

All state-level calculations are performed offline when constructing the REC–fidelity lookup table. The lookup table is generated over the initial-fidelity interval $F_{\textrm{initial}}\in [0.8,1.0]$ and is indexed by

43 $$\begin{aligned} \left( F_{\textrm{initial}}, \frac{\tau }{T_{1}}, M, X \right) , \end{aligned}$$

where *M* is the number of successful BBPSSW purification rounds and *X* is the number of elementary links. For each candidate path, $F_{\textrm{initial}}$ is set to the conservative effective value defined in Eq. (12).

Consequently, no direct coherence measurement is performed on an entangled pair used for communication. The model evaluates initial state imperfections, amplitude damping, twirling, BBPSSW purification, entanglement swapping, fidelity, and REC consistently within the same density-matrix framework.

### Network and request model

In this study, the quantum network is modeled as a graph *G=(V,E,C)*, where *V* denotes the set of quantum nodes, *E* denotes the set of quantum links, and *C* denotes the corresponding channel capacities. For each link *e=(i,j)∈ E*, the available capacity is denoted by *c*(*i*, *j*). Routing is performed by a centralized controller under an on-demand operation model.

Each connection request is defined as

44 $$\begin{aligned} r= \left( s_r, t_r, d_{x,r}, F_{\textrm{th},r}, a_r, b_r \right) , \end{aligned}$$

where $s_r$ and $t_r$ denote the source and destination nodes, respectively, $d_{x,r}$ denotes the traffic demand, $F_{\textrm{th},r}$ denotes the required end-to-end fidelity threshold for the request, and $[a_r,b_r]$ denotes the activity interval of the request. For each incoming request *r*, an edge-disjoint candidate-path set is constructed as

45 $$\begin{aligned} {\mathscr {P}}_r = \left\{ P_{r,j} \right\} . \end{aligned}$$

In the traffic model, a total of $N_{\textrm{req}}$ requests are generated within an arrival window of duration $T_{\textrm{win}}$. The arrival time of each request is independently drawn according to

46 $$\begin{aligned} a_r \sim {\mathscr {U}} \left( 0, T_{\textrm{win}} \right) . \end{aligned}$$

The departure time $b_r$ satisfies $b_r>a_r$, and the interval $[a_r,b_r]$ determines the period during which the request occupies the allocated network resources. Requests whose activity intervals overlap may therefore compete for the capacities of shared quantum links. Hence, $T_{\textrm{win}}$ controls the temporal distribution of the generated requests and the resulting level of traffic concurrency.

For a candidate path *P*, the end-to-end processing time is defined as

47 $$\begin{aligned} T(P) = T_{\textrm{ent}}(P) + T_{\textrm{pur}}(P) + T_{\textrm{swap}}(P), \end{aligned}$$

where $T_{\textrm{ent}}(P)$, $T_{\textrm{pur}}(P)$, and $T_{\textrm{swap}}(P)$ denote the entanglement-generation, purification, and entanglement-swapping times, respectively. A candidate path is temporally feasible only if

48 $$\begin{aligned} T(P) \le T_{\textrm{th}}, \end{aligned}$$

where $T_{\textrm{th}}$ is the maximum processing time allowed for one request. Thus, $T_{\textrm{win}}$ determines the traffic intensity, whereas $T_{\textrm{th}}$ determines the temporal feasibility of an individual request.

The routing strategies examined in this study rely on the same network setups, request sets, candidate paths, physical noise model, amplitude-damping and BBPSSW lookup table, and E2E state-evaluation process. They differ in when the REC is added to the routing and request-evaluation process.

In CAFARA, the E2E REC is used when determining purification levels, checking candidate-path feasibility, selecting paths, and validating final requests. Thus, CAFARA chooses the lowest purification level that meets the requirements for fidelity, coherence, capacity, and latency at the same time.

In FARA-PostREC, purification planning and candidate-path selection occur without using the REC. The necessary purification level is set based on fidelity, capacity, and latency limits. However, once a path is chosen, the E2E REC is assessed and added to the final request-success criteria.

In FARA-NoREC, the REC is not involved at any stage—neither in purification planning, candidate-path selection, nor final request evaluation. Therefore, this method provides a complete fidelity-only routing and purification baseline.

Accordingly, a request processed by CAFARA or FARA-PostREC is considered successful only if its selected path satisfies

49 $$\begin{aligned} T(P_r)\le & T_{\textrm{th}}, \end{aligned}$$

50 $$\begin{aligned} F_{\textrm{E2E}}(P_r)\ge & F_{\textrm{th},r}, \end{aligned}$$

and

51 $$\begin{aligned} C_{\textrm{rel,E2E}}(P_r) \ge C_{\textrm{rel}}^{\textrm{th}}, \end{aligned}$$

where $F_{\textrm{E2E}}(P_r)$ and $C_{\textrm{rel,E2E}}(P_r)$ denote the final E2E fidelity and REC of the selected path, respectively.

For FARA-NoREC, the final request-success condition contains only the latency and fidelity requirements:

52 $$\begin{aligned} T(P_r) \le T_{\textrm{th}}, \end{aligned}$$

and

53 $$\begin{aligned} F_{\textrm{E2E}}(P_r) \ge F_{\textrm{th},r}. \end{aligned}$$

In all routing strategies, the chosen path must meet the capacity, raw Bell-pair, entanglement-generation rate, and admission requirements before processing the request. This separation enables the numerical evaluation to identify three different effects: completely ignoring coherence information, using coherence solely as a post-selection quality constraint, and actively including coherence in purification planning and routing choices.

The average latency reported in the Results section is computed only over successfully served requests. Let ${\mathscr {R}}_{\textrm{succ}}$ denote the set of requests satisfying the final success criterion of the corresponding routing strategy. For each successful request *r*, let $P_r$ denote its selected path and let $T(P_r)$ denote its end-to-end processing time. The average latency of successful requests is defined as

54 $$\begin{aligned} \textrm{AL} = \frac{1}{|{\mathscr {R}}_{\textrm{succ}}|} \sum _{r\in {\mathscr {R}}_{\textrm{succ}}} T(P_r). \end{aligned}$$

Requests that do not satisfy the final success criterion are excluded from the latency calculation.

### Path feasibility and resource constraints

For a candidate path to be considered feasible, an S-D connection must exist, the quality of the states to be delivered must be ensured, and the latency and resource limits provided by the path must also be satisfied simultaneously. Let $P=(v_0,v_1,\ldots ,v_m)$ represent a candidate path, where $v_0$ is the source node, $v_m$ is the destination node, $v_i$ is the *i*-th node on the path, and *m* is the number of hops. The collection of intermediate nodes on path *P* is defined as

55 $$\begin{aligned} I(P)=\{v_1,v_2,\ldots ,v_{m-1}\}. \end{aligned}$$

The usable capacity of path *P*, represented as *U*(*P*), is defined by considering both the bottleneck link capacity and the success probabilities of entanglement swapping at the intermediate nodes. Therefore, the usable capacity is calculated as^37^.

56 $$\begin{aligned} U(P)= \left( \min _{0\le i<m} c(v_i,v_{i+1}) \right) \left( \prod _{u\in I(P)} p_{\textrm{swap}}(u) \right) , \end{aligned}$$

where $c(v_i,v_{i+1})$ denotes the available capacity of the quantum link between two consecutive nodes $v_i$ and $v_{i+1}$. $p_{\textrm{swap}}(u)$ denotes the entanglement-swapping success probability assigned to intermediate node *u*.

The first term in Eq. (56) represents the bottleneck capacity along the path. The second term shows the probability that all required swapping operations along the path are completed. Unlike the nominal channel capacity, *U(P)* represents the effective usable capacity after considering the probabilistic nature of entanglement swapping.

For each candidate path, the purification level is chosen from the set.

57 $$\begin{aligned} {\mathscr {M}} = \{0,1,\ldots ,M_{\max }\}. \end{aligned}$$

where *M=0* indicates the case where no purification is applied. For each purification level $M\in {\mathscr {M}}$, the lookup-table evaluation gives the end-to-end fidelity $F_{\textrm{E2E}}(P,M)$ and the end-to-end relative entropy of coherence $C_{\textrm{rel,E2E}}(P,M)$.

The corresponding end-to-end processing time is

58 $$\begin{aligned} T(P,M)= T_{\textrm{ent}}(P) + T_{\textrm{pur}}(P,M) + T_{\textrm{swap}}(P), \end{aligned}$$

where $T_{\textrm{ent}}(P)$, $T_{\textrm{pur}}(P,M)$, and $T_{\textrm{swap}}(P)$ represent the times for generating entanglement, purifying, and swapping entanglement, respectively.

The number of raw Bell pairs needed to fulfill a request *r* on path *P* with purification level *M* is represented by

59 $$\begin{aligned} B^{\textrm{req}}(P,M,r), \end{aligned}$$

and is determined from the request demand and the lookup-based expected raw-pair consumption of the BBPSSW purification procedure.

The following four conditions are considered when evaluating a candidate path and purification level:

60 $$\begin{aligned} C_{\textrm{rel,E2E}}(P,M)\ge & C_{\textrm{rel}}^{\textrm{th}}, \end{aligned}$$

61 $$\begin{aligned} F_{\textrm{E2E}}(P,M)\ge & F_{\textrm{th},r}, \end{aligned}$$

62 $$\begin{aligned} T(P,M)\le & T_{\textrm{th}}, \end{aligned}$$

and

63 $$\begin{aligned} U(P) \ge B^{\textrm{req}}(P,M,r). \end{aligned}$$

Here, $C_{\textrm{rel}}^{\textrm{th}}$ represents the minimum required E2E REC. $F_{\textrm{th},r}$ indicates the E2E fidelity requirement for request *r*. $T_{\textrm{th}}$ is the maximum allowable processing time for each request. $B^{\textrm{req}}(P,M,r)$ denotes the required number of raw Bell pairs.

The routing strategies vary based on where the REC condition in Eq. (60) is applied. Table 2 outlines the constraints utilized by CAFARA, FARA-PostREC, and FARA-NoREC during purification planning, checking the feasibility of candidate paths, and validating final requests.

**Table 2:** Use of relative entropy of coherence at different stages of the routing strategies.

Strategy	REC in purification	REC in path selection	REC in final success
CAFARA	Yes	Yes	Yes
FARA-PostREC	No	No	Yes
FARA-NoREC	No	No	No

For CAFARA, the set of feasible purification levels for candidate path *P* and request *r* is defined as

64 $$\begin{aligned} {\mathscr {M}}_{\textrm{CAFARA}}(P,r) = \left\{ M\in {\mathscr {M}} \; \bigg |\; \begin{array}{l} C_{\textrm{rel,E2E}}(P,M) \ge C_{\textrm{rel}}^{\textrm{th}},\\ F_{\textrm{E2E}}(P,M) \ge F_{\textrm{th},r},\\ T(P,M) \le T_{\textrm{th}},\\ U(P) \ge B^{\textrm{req}}(P,M,r) \end{array} \right\} . \end{aligned}$$

A candidate path is feasible for CAFARA only if

65 $$\begin{aligned} {\mathscr {M}}_{\textrm{CAFARA}}(P,r) \ne \varnothing . \end{aligned}$$

If this set is nonempty, CAFARA selects the minimum feasible purification level:

66 $$\begin{aligned} M_{\textrm{CAFARA}}^{\star }(P,r) = \min {\mathscr {M}}_{\textrm{CAFARA}}(P,r). \end{aligned}$$

Thus, CAFARA uses REC proactively during purification-level determination and candidate-path feasibility checking. A path that does not meet the REC requirement may become feasible through a higher purification level. This is provided with the resulting raw Bell-pair requirement, and latency remains within the available resource and time limits.

In both FARA-PostREC and FARA-NoREC, REC is not used during purification-level determination or candidate-path feasibility checking. Therefore, the two FARA variants use the same feasible purification-level set:

67 $$\begin{aligned} {\mathscr {M}}_{\textrm{FARA}}(P,r) = \left\{ M\in {\mathscr {M}} \;\bigg |\; \begin{array}{l} F_{\textrm{E2E}}(P,M) \ge F_{\textrm{th},r},\\ T(P,M) \le T_{\textrm{th}},\\ U(P) \ge B^{\textrm{req}}(P,M,r) \end{array} \right\} . \end{aligned}$$

A candidate path is feasible for either FARA variant only if

68 $$\begin{aligned} {\mathscr {M}}_{\textrm{FARA}}(P,r) \ne \varnothing , \end{aligned}$$

and the minimum feasible purification level is selected as

69 $$\begin{aligned} M_{\textrm{FARA}}^{\star }(P,r)=\min {\mathscr {M}}_{\textrm{FARA}}(P,r). \end{aligned}$$

Consequently, FARA-PostREC and FARA-NoREC perform identical purification planning and candidate-path feasibility checking. They therefore select the same purification level and evaluate the same set of feasible candidate paths under the same network and request realization. Their difference appears during the final request-success validation. After a path has been selected, FARA-PostREC additionally requires

70 $$\begin{aligned} C_{\textrm{rel,E2E}} \left( P, M_{\textrm{FARA}}^{\star }(P,r) \right) \ge C_{\textrm{rel}}^{\textrm{th}}, \end{aligned}$$

whereas FARA-NoREC does not apply a REC threshold during final request validation.

For CAFARA and FARA-PostREC, a request is therefore successful only if the selected path satisfies the final fidelity, REC, and latency requirements. For FARA-NoREC, the final success decision is based only on fidelity and latency, since REC is excluded from both routing and request validation. Among the feasible paths, all three routing strategies select the candidate path with the highest rate of entanglement generation. Therefore, the difference among the three strategies originates from the stage at which REC is incorporated, rather than from the final EGR-based path-ranking rule.

### Purification planning for FARA and CAFARA

For each request *r* and candidate path *P*, purification planning is performed by evaluating the purification levels defined in Eq. (57) in ascending order. Here, *M=0* corresponds to transmission without purification, whereas *M\ge 1* denotes *M* successful BBPSSW purification rounds. For each candidate path and purification level *M*, the final E2E fidelity and REC are obtained directly from the precomputed lookup table. Let *X(P)* denote the number of elementary links on candidate path *P*. When the elementary links of a path have different initial fidelities, the conservative effective initial fidelity is defined as in Eq. (12).

For every purification level $M\in {\mathscr {M}}$, the lookup table is queried using the effective path-level parameters

71 $$\begin{aligned} \left( F_{\textrm{initial}}^{P}, \frac{\tau ^{P}}{T_1}, M, X(P) \right) , \end{aligned}$$

where $F_{\textrm{initial}}^{P}$ is the minimum initial fidelity among the elementary links of candidate path *P*, $\tau ^{P}=T_{\textrm{ent}}(P)$ is the effective path-level amplitude-damping exposure, and *X*(*P*) is the number of elementary links in the path.

The lookup operation directly returns the corresponding final E2E fidelity, E2E REC, and expected raw-pair factor:

72 $$\begin{aligned} \bigl (F_{\textrm{E2E}}(P,M), C_{\textrm{rel,E2E}}(P,M), R_{\textrm{raw}}(P,M)\bigr ) = D\!\left( F_{\textrm{initial}}^{P}, \frac{\tau ^{P}}{T_1}, M, X(P)\right) . \end{aligned}$$

Thus, fidelity and REC are evaluated directly for every candidate purification level. Let $F_k(P)$ denote the link-level fidelity at the input of BBPSSW purification level *k*. The success probability of this purification level is

73 $$\begin{aligned} q_k(P) = P_{\textrm{BBPSSW}} \left( F_k(P) \right) , \end{aligned}$$

where $P_{\textrm{BBPSSW}}(\cdot )$ is the BBPSSW success-probability expression defined previously.

Each BBPSSW operation consumes two input Bell pairs and retains one output pair only when the purification attempt succeeds. Therefore, an unsuccessful attempt requires the two input pairs to be generated again. The expected number of raw elementary Bell pairs required to obtain one surviving pair after *M* purification levels is recursively defined as

74 $$\begin{aligned} R_{\textrm{raw}}(P,0)=1, \end{aligned}$$

and

75 $$\begin{aligned} R_{\textrm{raw}}(P,k+1) = \frac{ 2R_{\textrm{raw}}(P,k) }{ q_k(P) }. \end{aligned}$$

Equivalently, for *M\ge 1*,

76 $$\begin{aligned} R_{\textrm{raw}}(P,M) = \prod _{k=0}^{M-1} \frac{ 2 }{ P_{\textrm{BBPSSW}} \left( F_k(P) \right) } = \frac{ 2^M }{ \displaystyle \prod _{k=0}^{M-1} P_{\textrm{BBPSSW}} \left( F_k(P) \right) }. \end{aligned}$$

The quantity $R_{\textrm{raw}}(P,M)$ represents the expected raw-pair consumption required to produce one purified Bell pair at purification depth *M*. In this expression, $F_k(P)$ denotes the Werner-state fidelity of candidate path *P* at the input of the *k*-th BBPSSW purification round. The deterministic factor $2^M$ accounts for the exponential Bell-pair consumption at each purification level, while the denominator accounts for the expected repetitions caused by unsuccessful purification attempts, whose probabilities depend on the intermediate fidelities $F_k(P)$^27,35,38^.

If all purification attempts are assumed to succeed, i.e.,

77 $$\begin{aligned} P_{\textrm{BBPSSW}} \left( F_k(P) \right) =1, \qquad \forall k, \end{aligned}$$

then Eq. (76) reduces to

78 $$\begin{aligned} R_{\textrm{raw}}(P,M)=2^M, \end{aligned}$$

which corresponds to the deterministic purification-overhead term used in the previous resource-demand formulation^38^.

The raw-pair factor in Eq. (76) corresponds to the production of one purified Bell pair and does not yet include the request demand. Let $d_{x,r}$ denote the number of end-to-end entangled pairs requested by request *r*. The required raw Bell-pair demand for request *r*, candidate path *P*, and purification level *M* is therefore

79 $$\begin{aligned} B_{\textrm{req}}(P,M,r) = \lceil d_{x,r} R_{\textrm{raw}}(P,M) \rceil . \end{aligned}$$

Substituting Eq. (76) into Eq. (79) gives

80 $$\begin{aligned} B_{\textrm{req}}(P,M,r) = \big \lceil \frac{ d_{x,r}\,2^M }{ \displaystyle \prod _{k=0}^{M-1} P_{\textrm{BBPSSW}} \left( F_k(P) \right) } \big \rceil . \end{aligned}$$

Equation (80) generalizes the deterministic resource-demand model

81 $$\begin{aligned} B_{\textrm{req}}^{\textrm{det}}(P,M,r) = \lceil d_{x,r}\,2^M \rceil . \end{aligned}$$

by accounting for the expected repetitions caused by unsuccessful BBPSSW purification attempts.

The entanglement-swapping success factor is already included in the usable capacity definition in Eq. (56). Therefore, the capacity-feasibility condition is evaluated using Eq. (63), and the swapping-success factor is not included again in $B_{\textrm{req}}(P,M,r)$.

If no purification is applied, *M=0*, the product over purification levels is defined as one as in Eq. 74

Consequently,

82 $$\begin{aligned} B_{\textrm{req}}(P,0,r) = \lceil d_{x,r} \rceil . \end{aligned}$$

For every purification level, the associated end-to-end processing time is computed using the latency model defined in Eq. (58).

The two FARA variants use the feasible purification-level set defined in Eq. (67) and select the minimum feasible level according to Eq. (69). Therefore, FARA-PostREC and FARA-NoREC use the same purification level and the same raw Bell-pair requirement for a given candidate path. Their difference appears only during final request validation, where FARA-PostREC additionally applies the REC threshold, whereas FARA-NoREC does not apply a coherence constraint.

CAFARA instead uses the feasible purification-level set defined in Eq. (64) and selects the minimum feasible level according to Eq. (66). Thus, CAFARA may select a higher purification level compared to FARA when the requested fidelity has already been achieved, but the E2E REC remains below the required threshold, provided that the corresponding raw Bell-pair demand and latency remain within the available resource and time limits. The purification levels are examined in ascending order. Therefore, the first level satisfying the algorithm-specific constraints is the selected minimum feasible purification level. If no purification level satisfies the corresponding conditions, the candidate path is declared infeasible.

Consequently, purification planning is treated jointly as a state-quality, latency, and capacity-allocation problem. For each candidate path, the routing algorithm determines the minimum feasible purification level, the associated expected raw Bell-pair demand, and the resulting E2E fidelity and REC before the final EGR-based path-selection step is performed.

### Routing workflow

According to our proposed routing procedure, a set of link-disjoint candidate paths is first generated between the source and destination nodes for each incoming request *r*. For each candidate path *P*, the number of elementary links *X(P)*, usable capacity *U(P)*, entanglement generation time, purification time, entanglement-swapping time, E2E fidelity, E2E REC, and EGR are calculated and evaluated. Purification planning is performed by finding the purification levels defined in Eq. (57) and arranging them in ascending order. For each $M\in {\mathscr {M}}$, the precomputed amplitude-damping–BBPSSW lookup table directly gives $F_{\textrm{E2E}}(P,M)$, $C_{\textrm{rel,E2E}}(P,M)$, and the expected raw-pair factor value associated with the selected purification depth. Then, we obtain the required number of raw Bell pairs using Eq. (79).

The selected routing strategy determines the feasibility conditions for purification. CAFARA applies the feasible purification-level set defined in Eq. (64). In Eq. (64), the decision jointly depends on the fidelity, REC, latency, and capacity constraints. In contrast, both FARA variants use the feasible set defined in Eq. (67), where REC is discarded from purification-level determination and candidate-path feasibility checking. $C_{\textrm{rel,E2E}}(P,M)$ is obtained from the lookup table for reporting and final validation of FARA variants as well. However, it does not affect purification planning or candidate-path feasibility in either FARA variant.

First, the minimum feasible purification level is determined for each candidate path. Then, paths with insufficient usable capacity, excessive latency, unacceptable state quality under the corresponding routing strategy, or non-positive EGR are discarded. Among the remaining feasible paths, the path having the highest EGR is selected:

83 $$\begin{aligned} P_r^\star = \arg \max _{P\in {\mathscr {P}}_r^{\textrm{feasible}}} \textrm{EGR}(P). \end{aligned}$$

The selected path is then processed through elementary entanglement generation, link-level BBPSSW purification when $M^\star >0$, and entanglement swapping at the intermediate nodes. The final E2E fidelity, REC, and processing latency are subsequently evaluated using the selected path and purification level information.

If the selected path satisfies the final fidelity, REC, and latency requirements given in Eqs. (49–51), a request is counted as successful for CAFARA. FARA-PostREC uses the same fidelity-based purification and path-selection procedure with FARA-NoREC, but it additionally applies the REC threshold during final request validation. In contrast, FARA-NoREC does not apply an REC constraint during purification planning, path selection, or final success evaluation. Its final success criterion is therefore limited to the latency and fidelity requirements given in Eqs. (52–53). If any strategy-specific condition is violated, the request is rejected, and it is assigned to the corresponding drop reason.

**Fig. 6:**
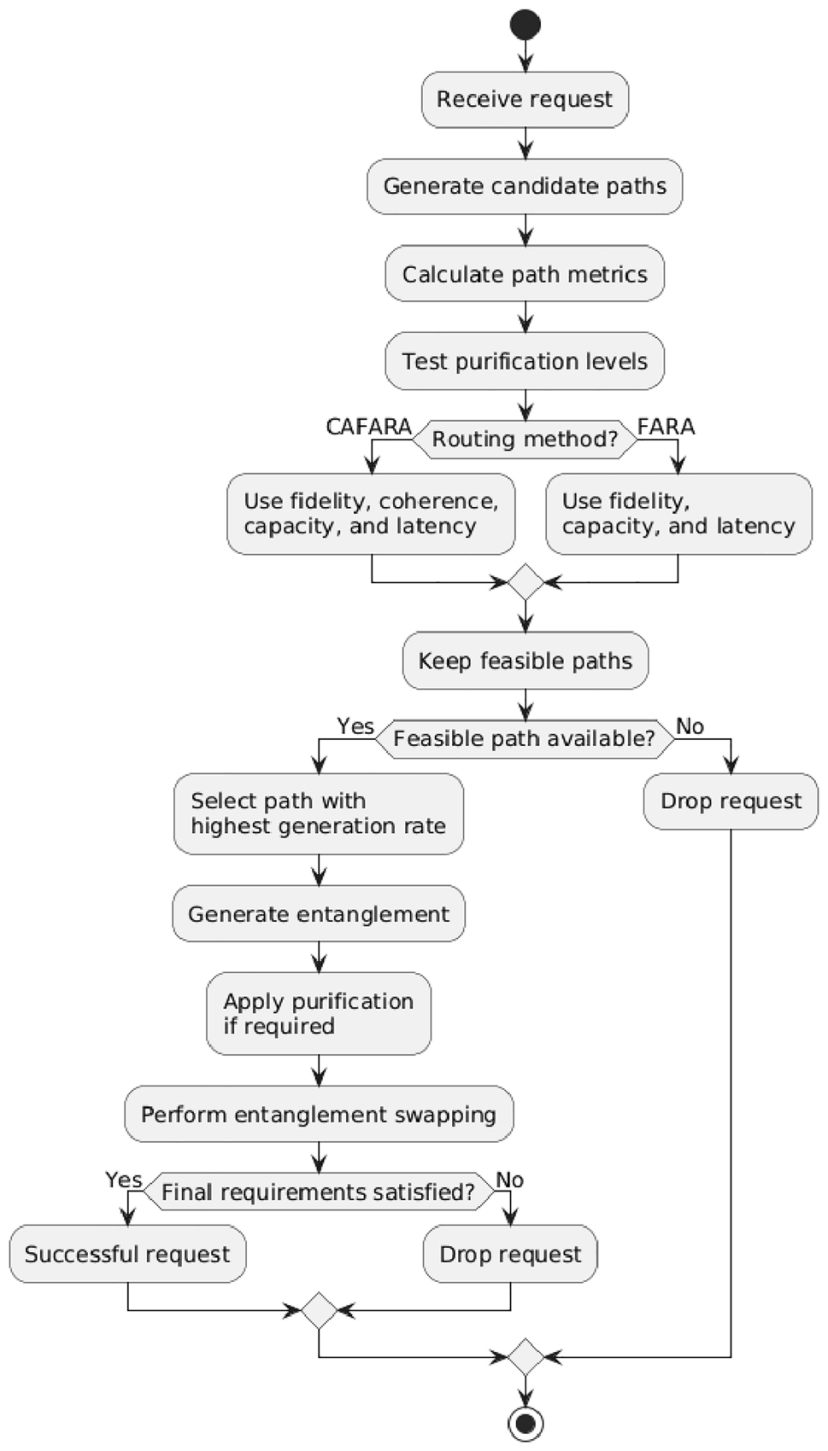
Overview of the routing workflow for CAFARA, FARA-PostREC, and FARA-NoREC. For each request, edge-disjoint candidate paths are generated and evaluated based on usable capacity, latency, end-to-end fidelity, end-to-end REC, raw Bell-pair demand, and EGR. For every candidate path, purification levels are examined in ascending order. CAFARA selects the minimum level satisfying the fidelity, REC, latency, and capacity constraints, whereas both FARA variants determine the purification level without using REC. Among the feasible candidate paths, the path with the highest positive EGR is selected. FARA-PostREC applies the REC threshold only during final request validation, while FARA-NoREC does not apply an REC constraint.

In all three strategies, the selected path must also satisfy the admission and resource-reservation conditions (Fig. 6).

#### Entanglement generation rate

The EGR^39^ is defined as the inverse of the expected E2E entanglement generation time of a candidate path and is used as the final selection metric among feasible candidate paths. At the link level, the EGR of a link is defined by taking quantum-memory and coherence feasibility into account as^39^

84 $$\begin{aligned} EGR_{i,j}(T_{ch})= {\left\{ \begin{array}{ll} 0, & T_{ch}<\xi _{i,j},\\ \dfrac{1}{T^{\textrm{link}}_{i,j}}, & \text {otherwise,} \end{array}\right. } \end{aligned}$$

where $T_{ch}$ denotes the quantum-memory coherence time, $\xi _{i,j}$ denotes the minimum storage-time requirement for the corresponding link, and $T^{\textrm{link}}_{i,j}$ denotes the expected time required to generate one elementary entangled pair.

At the path level, let the candidate path be denoted by $P_{r,j}=(v_0,v_1,\ldots ,v_m)$, where *m* is the number of elementary links. The expected end-to-end entanglement generation time $T^{EGR}(P_{r,j})$ is computed recursively by jointly accounting for the generation times of subpaths, the Bell-state measurement duration, and classical signaling delays. Accordingly, the EGR of the path is obtained as^39^

85 $$\begin{aligned} EGR(P_{r,j};T_{ch})= {\left\{ \begin{array}{ll} 0, & T_{ch} < \xi (P_{r,j}) - \min \limits _{l=0,\dots ,m-1} \left( T^s_{v_l,v_{l+1}} - \xi _{v_l,v_{l+1}} \right) , \\ \dfrac{1}{T^{EGR}(P_{r,j})}, & \text {otherwise.} \end{array}\right. } \end{aligned}$$

Here, $\xi (P_{r,j})$ denotes the minimum path-level storage-time requirement for successfully establishing end-to-end entanglement over $P_{r,j}$. For the *l*-th elementary link $(v_l,v_{l+1})$, $T^s_{v_l,v_{l+1}}$ denotes the average duration of a successful elementary link-entanglement attempt, whereas $\xi _{v_l,v_{l+1}}$ denotes the corresponding link-level storage-time requirement. The minimization is taken over all elementary links of candidate path $P_{r,j}$ and identifies the most restrictive link-level timing offset. This offset determines the worst-case memory-coherence requirement that must be satisfied for the path to have a positive EGR.

In this way, only paths that are feasible in terms of memory and coherence obtain a positive EGR value, and among the candidate paths satisfying the capacity, fidelity, REC, and time constraints, the one with the highest EGR is selected. In addition, the EGR model used in this work does not explicitly include purification delay; therefore, the effect of purification is considered separately in the latency evaluation.

## Supplementary Information

Supplementary Information.

## Data Availability

The simulation code used in this study is publicly available in the GitHub repository at https://github.com/hlltnc/CAFARA_FARA_FINAL_MAJOR-REVISION. The precomputed fidelity–coherence lookup table required by the CAFARA implementation is provided separately through Zenodo at https://doi.org/10.5281/zenodo.21338965.
