# Supplementary material for: Coherence and fidelity aware routing in quantum networks
Source: Sci Rep. 2026 Aug 6;16:24346. doi: 10.1038/s41598-026-65444-1 (PMC13448665; doi:10.1038/s41598-026-65444-1)
Supplement: Supplementary file 1 [file 41598_2026_65444_MOESM1_ESM.pdf]

Supplementary Information for
"Coherence and Fidelity Aware Routing in Quantum Networks"

Hilal Sultan Duranoglu Tunc[1,2,*], Joy Halder[3], Azita Hajizade[1,2], Andreas Voigt[1,2], Bassem Arar[1,2], Riccardo Bassoli[1,2,4], Gerhard P. Fettweis[3,4], and Frank H. P. Fitzek[1,4]

[1] Deutsche Telekom Chair of Communication Networks, Technische Universität Dresden, Dresden, Germany
[2] Quantum Communication Networks Research Group, Technische Universität Dresden, Dresden, Germany
[3] Vodafone Chair Mobile Communications Systems, Technische Universität Dresden, Dresden, Germany
[4] Centre for Tactile Internet with Human-in-the-Loop, Technische Universität Dresden, Dresden, Germany
[*] Correspondence to: hilal_sultan.duranoglu_tunc@tu-dresden.de

Contents

1 Supplementary Notation and Definitions **1**
1.1 Mathematical notation 1

2 Detailed Latency Model **7**
2.1 Overview of the Latency Components 7
2.2 Entanglement Generation Time 7
2.3 Entanglement Purification Time 8
2.4 Entanglement Swapping Time 9
2.5 E2E Latency and Average Latency 10

3 Detailed Routing Algorithms **11**
3.1 FARA-NoREC Routing Algorithm 11
3.2 FARA-PostREC Routing Algorithm 12
3.3 CAFARA Routing Algorithm 14

4 Time Complexity Analysis **16**
4.1 Time Complexity of the FARA-NoREC Routing Algorithm 17
4.2 Time Complexity of the FARA-PostREC Routing Algorithm 22
4.3 Time Complexity of the CAFARA Routing Algorithm 22

1 Supplementary Notation and Definitions

1.1 Mathematical notation

This section summarizes the mathematical notation used in the Supplementary Information. The notation is kept consistent with the main manuscript. Additional symbols introduced for the latency, purification, lookup table, and time-complexity analyses are also defined here.

Table S1: Supplementary mathematical notation used in the physical-state, routing, purification, latency, performance metric, lookup-table, and complexity analyses.

Symbol	Definition
Network and request model	
$G = (V, E, C)$	Quantum network graph, where V is the set of quantum nodes, E is the set of quantum links, and C denotes the set of link-capacity values.
$N = \|V\|$	Number of quantum nodes in the network.
$\|E\|$	Number of quantum links in the network.
$e = (i, j) \in E$	Quantum link between adjacent nodes i and j.
$c(i, j)$	Available capacity of quantum link (i, j).
$p_{\text{swap}}(u)$	Entanglement-swapping success probability assigned to intermediate node u.
$\text{ESP}(P)$	Cumulative entanglement-swapping success factor of path P, defined as the product of the swapping probabilities of its intermediate nodes.
r	Connection-request index.
N_{req}	Total number of generated connection requests.
$r = (s_r, t_r, d_{x,r}, F_{\text{th},r}, a_r, b_r)$	Connection request, where s_r and t_r are the source and destination nodes, $d_{x,r}$ is the requested number of end to end (E2E) Bell pairs, $F_{\text{th},r}$ is the required E2E fidelity, and $[a_r, b_r]$ is the activity interval.
s_r	Source node of request r.
t_r	Destination node of request r.
$d_{x,r}$	Traffic demand of request r, expressed as the requested number of E2E Bell pairs.
$F_{\text{th},r}$	Minimum E2E fidelity required by request r.
a_r	Arrival time of request r.
b_r	Departure time of request r.
$h_r = b_r - a_r$	Holding time of request r.
T_{win}	Time window within which the requests are generated.
T_{th}	Maximum allowable E2E processing time for one request.
Candidate paths and resource feasibility	
$\mathcal{P}_r = \{P_{r,j}\}$	Set of edge-disjoint candidate paths constructed for request r.
$P_{r,j}$	The j-th candidate path of request r.
$P = (v_0, v_1, \ldots, v_X)$	Generic path containing X elementary links, where v_0 is the source and v_X is the destination.
$X(P)$	Number of elementary links, or hop count, of path P.
X	Hop-count index used in the state-evolution lookup table.
$I(P)$	Set of intermediate nodes on path P.
$L_{v_k, v_{k+1}}$	Physical distance between two consecutive nodes on path P.
$U(P)$	Effective usable capacity of path P after accounting for the bottleneck link capacity and cumulative swapping-success factor.
$\mathcal{P}_r^{\text{feasible}}$	Set of candidate paths satisfying the strategy-specific quality, capacity, latency, and positive-EGR conditions for request r.
$P_r^{\star}$	Path selected for request r from the feasible candidate paths.
Quantum-state and amplitude-damping model	

**Symbol**	**Definition**
$\mathcal{B}$	Fixed two-qubit computational basis $\{\lvert 00\rangle, \lvert 01\rangle, \lvert 10\rangle, \lvert 11\rangle\}$ used to evaluate REC.
$\lvert\Phi^+\rangle$	Target maximally entangled Bell state, $\lvert\Phi^+\rangle = (\lvert 00\rangle + \lvert 11\rangle)/\sqrt{2}$.
ρ_{Φ^+}	Density matrix $\lvert\Phi^+\rangle\langle\Phi^+\rvert$.
ρ	Density matrix of a quantum state.
ρ_{diag}	Completely dephased state obtained by removing the off-diagonal entries of ρ in the computational basis.
$S(\rho)$	von Neumann entropy of ρ, $S(\rho) = -\operatorname{Tr}[\rho \log_2 \rho]$.
$C_{\text{rel}}(\rho)$	Relative entropy of coherence of state ρ, defined as $S(\rho_{\text{diag}}) - S(\rho)$.
$F_{\text{initial}}^{(\ell)}$	Initial fidelity assigned to elementary link ℓ.
F_{initial}^{P}	Conservative effective initial fidelity of path P, defined as $\min_{\ell \in P} F_{\text{initial}}^{(\ell)}$.
$\rho_{\text{initial}}(F_{\text{initial}})$	Imperfect initial Werner state having fidelity F_{initial} with respect to $\lvert\Phi^+\rangle$.
T_1	Characteristic energy-relaxation time of the amplitude-damping channel.
τ	Exposure duration used in the amplitude-damping model and lookup-table query.
τ/T_1	Normalized exposure duration used as a lookup-table index.
$\gamma(\tau)$	Amplitude-damping probability, $\gamma(\tau) = 1 - \exp(-\tau/T_1)$.
$E_0(\gamma), E_1(\gamma)$	Kraus operators of the one-sided amplitude-damping channel.
$\rho_{\text{AD}}(\tau; F_{\text{initial}})$	Elementary-link density matrix after one-sided amplitude damping.
$F_{\text{AD}}(\tau; F_{\text{initial}})$	Fidelity of the amplitude-damped elementary-link state.
$C_{\text{rel}}^{\text{AD}}(\tau; F_{\text{initial}})$	REC of the amplitude-damped elementary-link state.
Twirling, BBPSSW purification, and swapping	
$\rho_{\text{W}}(F)$	Werner state having fidelity F with respect to $\lvert\Phi^+\rangle$.
F_k	Werner-state fidelity at the input of purification level k.
$P_{\text{BBPSSW}}(F_k)$	Success probability of one BBPSSW [1] purification operation with two identical Werner-state inputs of fidelity F_k.
F_{k+1}	Conditional output fidelity after a successful BBPSSW operation at level k.
M	Number of successful BBPSSW [1] purification levels applied to each elementary link.
$M_{\max}$	Maximum purification level considered by the routing algorithms.
$\mathcal{M} = \{0, 1, \ldots, M_{\max}\}$	Set of candidate purification levels, where $M = 0$ denotes no purification.
$\text{Swap}(\rho_{AB_1}, \rho_{B_2C})$	Density-matrix operation describing entanglement swapping between two adjacent entangled states.
$\mathcal{B}_{\text{id}}$	Set of successfully identified Bell-state-measurement outcomes.
$\rho_{\text{E2E}}^{(M,X)}$	Final E2E density matrix after purification level M and $X-1$ entanglement-swapping operations.
$F_{\text{E2E}}(P, M)$	Final E2E fidelity predicted for path P at purification level M.
$C_{\text{rel,E2E}}(P, M)$	Final E2E REC predicted for path P at purification level M.
$C_{\text{rel}}^{\text{th}}$	Minimum acceptable E2E REC threshold.

Symbol	Definition
Lookup table and purification planning	
D	Precomputed amplitude-damping–BBPSSW lookup table.
$\left(F^{P}_{\text{initial}}, \tau/T_1, M, X(P)\right)$	Lookup-table index tuple for candidate path P and purification level M.
$R_{\text{raw}}(P, M)$	Expected number of raw elementary Bell pairs required to produce one surviving pair after M BBPSSW purification levels.
$q_k(P)$	BBPSSW success probability at purification level k on path P, $q_k(P) = P_{\text{BBPSSW}}(F_k(P))$.
$B_{\text{req}}(P, M, r)$	Expected number of raw Bell pairs required to satisfy request r on path P using purification level M.
$\mathcal{M}_{\text{CAFARA}}(P, r)$	Set of purification levels satisfying the fidelity, REC, latency, and capacity constraints for CAFARA.
$\mathcal{M}_{\text{FARA}}(P, r)$	Set of purification levels satisfying the fidelity, latency, and capacity constraints for FARA-PostREC and FARA-NoREC.
$M^{\star}_{\text{CAFARA}}(P, r)$	Minimum feasible purification level selected by CAFARA.
$M^{\star}_{\text{FARA}}(P, r)$	Minimum fidelity-based feasible purification level selected by FARA-PostREC and FARA-NoREC.
Latency and entanglement-generation rate	
$T(P, M)$	Total E2E processing time of path P at purification level M.
$T_{\text{ent}}(P)$	Elementary-entanglement-generation time of path P.
$T_{\text{pur}}(P, M)$	Purification time of path P at purification level M.
$T_{\text{swap}}(P)$	Entanglement-swapping and associated signaling time of path P.
c_{fiber}	Propagation speed of light in optical fiber.
T_{ch}	Quantum-memory time used in the EGR feasibility model.
$\xi_{i,j}$	Minimum storage-time requirement of link (i, j) in the EGR model.
$\xi(P_{r,j})$	Path-level storage-time requirement used in the EGR feasibility condition.
$T^{\text{link}}_{i,j}$	Expected time required to generate one elementary entangled pair over link (i, j).
$T^{\text{EGR}}(P_{r,j})$	Expected E2E entanglement-generation time used in the path-level EGR model.
$\text{EGR}_{i,j}(T_{\text{ch}})$	Link-level entanglement-generation rate under memory time T_{ch}.
$\text{EGR}(P_{r,j}; T_{\text{ch}})$	Entanglement-generation rate of candidate path $P_{r,j}$ under memory time T_{ch}.
Performance metrics	
$\mathcal{R}_{\text{succ}}$	Set of requests satisfying the final success criterion of the corresponding routing strategy.
ARSR	Average request success rate.
AL	Average E2E latency computed only over successfully served requests.
APT	Average purification time computed only over successfully served requests.
APL	Average physical path length computed only over successfully served requests.
AFF	Average final E2E fidelity computed only over successfully served requests.

Symbol	Definition
AFC	Average final E2E REC computed only over successfully served requests.
Time-complexity analysis	
R	Total number of connection requests considered in the time-complexity analysis.
$N = \|V\|$	Number of quantum nodes in the network graph.
$\|E\|$	Number of quantum links in the network graph.
$\mathcal{P}_r$	Set of edge-disjoint candidate paths generated for request r.
$n_r = \|\mathcal{P}_r\|$	Number of edge-disjoint candidate paths generated for request r.
$n_{\max}$	Maximum number of candidate paths generated for any request: $n_{\max} = \max_{1 \leq r \leq R} n_r$.
$H(P)$	Hop count of candidate path P, equivalently the number of elementary links in P, where $H(P) = \|P\| - 1$.
H_r	Maximum hop count among the candidate paths generated for request r: $H_r = \max_{P \in \mathcal{P}_r} H(P)$.
$H_{\max}$	Maximum candidate-path hop count over all requests: $H_{\max} = \max_{1 \leq r \leq R} H_r$.
$M_{\max} + 1$	Maximum number of purification levels examined for one candidate path, including the no-purification case $M = 0$.
D	Complete precomputed amplitude-damping–BBPSSW lookup table.
$\|D\|$	Total number of rows in the complete lookup table D.
K	Number of rows in the lookup-table subset associated with the selected source-fidelity value for a candidate path.
$K_{M,X}$	Number of rows in the selected source-fidelity subset corresponding to a fixed purification level M and hop count $X(P)$.
K_r	Maximum source-fidelity-subset size used while evaluating the candidate paths of request r.
$K_{\max}$	Maximum source-fidelity-subset size over all requests: $K_{\max} = \max_{1 \leq r \leq R} K_r$.
$\mathcal{P}_r^{\text{feasible}}$	Set of candidate paths of request r that satisfy the applicable purification-level feasibility conditions.
$f_r = \|\mathcal{P}_r^{\text{feasible}}\|$	Number of feasible candidate paths considered during overlap checking and maximum-EGR path selection for request r.
$\mathcal{A}_r$	Set of active reservations considered during the overlap check for request r.
$A_r = \|\mathcal{A}_r\|$	Number of active reservations considered during overlap checking for request r.
$A_{\max}$	Maximum number of active reservations considered for any request: $A_{\max} = \max_{1 \leq r \leq R} A_r$.

Table S2: Algorithm-specific variables and decision outcomes used in the CAFARA routing and purification procedure.

Symbol / Outcome	Definition
$G = (V, E, C)$	Quantum network graph, where V is the set of quantum nodes, E is the set of quantum links, and C denotes the currently available link capacities.
$r = (s_r, t_r, d_{x,r}, F_{\text{th},r}, a_r, b_r)$	Connection request with source s_r, destination t_r, traffic demand $d_{x,r}$, required E2E fidelity $F_{\text{th},r}$, arrival time a_r, and departure time b_r.
D	Precomputed amplitude-damping–BBPSSW lookup table used to obtain the predicted E2E fidelity, E2E REC, and raw Bell-pair requirement.
$\mathcal{P}_r$	Set of edge-disjoint candidate paths generated between s_r and t_r for request r.
P	A generic candidate path belonging to $\mathcal{P}_r$.
$X(P)$	Number of elementary links, or hop count, of candidate path P.
$U(P)$	Usable capacity of candidate path P, obtained from the bottleneck available link capacity and the swapping-success probabilities of its intermediate nodes.
F^P_{initial}	Conservative effective initial fidelity associated with candidate path P.
τ/T_1	Normalized amplitude-damping duration used in the lookup-table query, where $\tau = T_{\text{ent}}(P)$.
$\mathcal{M} = \{0, 1, \ldots, M_{\max}\}$	Set of candidate purification levels examined in ascending order, where $M = 0$ denotes an operation without purification.
$F_{\text{E2E}}(P, M)$	Predicted final E2E fidelity of candidate path P at purification level M.
$C_{\text{rel,E2E}}(P, M)$	Predicted final E2E relative entropy of coherence of candidate path P at purification level M.
$B_{\text{req}}(P, M, r)$	Expected number of raw Bell pairs required to serve request r over candidate path P using purification level M.
$T_{\text{ent}}(P)$	Entanglement-generation time of candidate path P.
$T_{\text{pur}}(P, M)$	Purification time of candidate path P at purification level M.
$T_{\text{swap}}(P)$	Entanglement-swapping time of candidate path P.
$T(P, M)$	Total E2E processing time of candidate path P at purification level M, defined as $T_{\text{ent}}(P) + T_{\text{pur}}(P, M) + T_{\text{swap}}(P)$.
$M^\star_P$	Minimum purification level satisfying the fidelity, REC, latency, and capacity constraints for candidate path P. It remains undefined if no such level exists.
$\text{EGR}(P)$	Entanglement generation rate of candidate path P. Only paths with $\text{EGR}(P) > 0$ are retained for final path selection.
$\mathcal{P}^{\text{feasible}}_r$	Set of candidate paths having a defined minimum feasible purification level $M^\star_P$ and a positive EGR.
$P^\star_r$	Path selected for request r according to the maximum-EGR rule: $$P^\star_r = \arg \max_{P \in \mathcal{P}^{\text{feasible}}_r} \text{EGR}(P).$$
$M^\star_r$	Purification level associated with the selected path, defined as $M^\star_r = M^\star_{P^\star_r}$.
Reject(*no path*)	Decision returned when no edge-disjoint source–destination candidate path can be generated, i.e., $\mathcal{P}_r = \emptyset$.
Reject(*no jointly feasible path*)	Decision returned when no candidate path has both a feasible purification level and a positive EGR, i.e., $\mathcal{P}^{\text{feasible}}_r = \emptyset$.
$\text{Reject}_{\text{final}}$	Decision returned when the selected path fails at least one final fidelity, REC, latency, or capacity requirement.
Successful	Decision returned when the selected path and purification level satisfy the final fidelity, REC, latency, and capacity requirements.

2 Detailed Latency Model

2.1 Overview of the Latency Components

In this work, the latency of a request is defined as the end-to-end processing time required to establish entanglement over the selected path. For a selected path P, this latency consists of three components: entanglement generation time, entanglement purification time, and entanglement swapping time. Therefore, the total latency is expressed as

$$T(P) = T_{\text{gen}}(P) + T_{\text{pur}}(P) + T_{\text{swap}}(P), \tag{S1}$$

where $T_{\text{gen}}(P)$ denotes the entanglement generation time, $T_{\text{pur}}(P)$ denotes the purification time, and $T_{\text{swap}}(P)$ denotes the entanglement swapping time.

For FARA and CAFARA, purification can be required to satisfy the target quality constraints. Hence, the full latency expression in Eq. (S1) is used. A selected path is considered time-feasible only if

$$T(P) \leq T_{\text{th}}, \tag{S2}$$

where T_{th} is the per-request latency threshold.

2.2 Entanglement Generation Time

Entanglement generation is the process of preparing two or more quantum systems in a joint quantum state that cannot be written as a product of individual states [2]. In this work, the term entanglement refers to the generation of Bell states, i.e., maximally entangled two-qubit states such as $|\Phi^+\rangle = (|00\rangle + |11\rangle)/\sqrt{2}$.

For an elementary quantum link, we compute the average entanglement generation time following [3]:

$$\langle T \rangle_0 = \frac{T_0}{p_{\text{success}}}, \tag{S3}$$

where

$$T_0 = \frac{2L_0}{c_{\text{fiber}}} \tag{S4}$$

is the round-trip signaling time over an elementary link of length L_0, and $c_{\text{fiber}} = 2 \times 10^8$ m/s is the speed of light in optical fiber. The term p_{success} denotes the probability of successful entanglement generation and is given by [3]

$$p_{\text{success}} = 1 - p_{\text{failure}}. \tag{S5}$$

The failure probability and the transmission parameter of the quantum channel are defined as [3]

$$p_{\text{failure}} = (2F - 1)^{\frac{\eta}{1-\eta}}, \qquad \eta(L_0, L_{\text{att}}) = e^{-\frac{L_0}{L_{\text{att}}}}, \tag{S6}$$

where F denotes the fidelity of the elementary entangled state, L_{att} is the attenuation length, and η represents the probability that a photon is successfully transmitted over the distance L_0. In our analysis, we set $L_{\text{att}} = L_0$, so that the attenuation length equals the elementary link distance.

For a candidate path $P = (v_0, v_1, \ldots, v_m)$, the path-level entanglement generation time is obtained by accumulating the elementary-link generation times:

$$T_{\text{gen}}(P) = \sum_{i=0}^{m-1} \langle T \rangle_0^{(v_i, v_{i+1})}. \tag{S7}$$

If all active links have the same physical length within a given experiment, this expression reduces to

$$T_{\text{gen}}(P) = X(P)\,\langle T\rangle_0, \tag{S8}$$

where $X(P)$ is the hop count of path P.

2.3 Entanglement Purification Time

Entanglement purification is an LOCC-based procedure that consumes multiple imperfect entangled pairs to produce a smaller number of pairs with improved entanglement quality [4]. In this work, purification is implemented using the BBPSSW protocol after the amplitude-damped elementary link state has been transformed into Werner form through twirling. The same BBPSSW model is used in the state-evolution lookup table, the raw Bell-pair requirement, and the purification-time calculation.

Let $F_k(P)$ denote the Werner-state fidelity at the input of purification level k for candidate path P. For two identical Werner-state inputs, the success probability of one BBPSSW operation is [1, 5]

$$q_k(P) = P_{\text{BBPSSW}}(F_k(P)) = F_k(P)^2 + \frac{2}{3}F_k(P)\,[1 - F_k(P)] + \frac{5}{9}\,[1 - F_k(P)]^2\,. \tag{S9}$$

Conditioned on a successful purification operation, the fidelity supplied to the next purification level is

$$F_{k+1}(P) = \frac{F_k(P)^2 + \frac{1}{9}\,[1 - F_k(P)]^2}{F_k(P)^2 + \frac{2}{3}F_k(P)\,[1 - F_k(P)] + \frac{5}{9}\,[1 - F_k(P)]^2}. \tag{S10}$$

The purification levels are applied sequentially because the output pair of level k is used as the input pair of level $k+1$. However, purification operations belonging to the same level are assumed to be performed in parallel on the elementary links of the candidate path.

Let $\ell = (v_i, v_{i+1})$ denote an elementary link of path P, and let L_ℓ be its physical length. The time associated with one BBPSSW level on link ℓ is modeled as

$$t^{(1)}_{\text{pur}}(\ell) = \frac{2L_\ell}{c_{\text{fiber}}} + t_{\text{loc}}, \tag{S11}$$

where $c_{\text{fiber}} = 2 \times 10^8$ m/s is the propagation speed in optical fiber and t_{loc} represents the local-operation and measurement time associated with one BBPSSW level. The term $2L_\ell/c_{\text{fiber}}$ represents the round-trip classical signaling time used to compare the local measurement outcomes and determine whether the purified pair is retained. This propagation-based timing assumption is consistent with the communication-delay treatment used in repeater-assisted purification analyses [3].

Because the elementary-link purification operations are executed in parallel, the duration of one path-level purification stage is determined by the slowest elementary link:

$$t^{(1)}_{\text{pur}}(P) = \max_{\ell\in P}\left\{\frac{2L_\ell}{c_{\text{fiber}}} + t_{\text{loc}}\right\}. \tag{S12}$$

For a candidate path P and a selected purification level M, the total purification contribution to the E2E latency is therefore

$$T_{\text{pur}}(P, M) = M\,\max_{\ell\in P}\left\{\frac{2L_\ell}{c_{\text{fiber}}} + t_{\text{loc}}\right\}. \tag{S13}$$

In the numerical evaluation, the local-operation term is set to $t_{\text{loc}} = 0$. Consequently, the implemented purification-time model reduces to

$$T_{\text{pur}}(P, M) = \frac{2M}{c_{\text{fiber}}}\max_{\ell\in P} L_\ell. \tag{S14}$$

When $M = 0$, no purification is performed and

$$T_{\text{pur}}(P, 0) = 0. \tag{S15}$$

The BBPSSW success probabilities are used separately to determine the expected raw Bell-pair consumption. Specifically, unsuccessful purification attempts increase the expected raw-pair factor $R_{\text{raw}}(P, M)$ and hence the required Bell-pair demand $B_{\text{req}}(P, M, r)$. They are not included again as an additional multiplicative delay in Eq. (S13); this avoids counting the same purification-failure overhead in both the resource-demand and purification-time models.

CAFARA, FARA-PostREC, and FARA-NoREC use the same purification-time model. Their purification times differ because the selected purification level is algorithm-dependent. CAFARA determines the minimum level satisfying the fidelity, REC, latency, and capacity constraints, whereas the two FARA variants determine the minimum level using the fidelity, latency, and capacity constraints only. Consequently, CAFARA may select a larger value of M when the fidelity threshold has already been reached, but the predicted E2E REC remains below $C_{\text{rel}}^{\text{th}}$.

2.4 Entanglement Swapping Time

Entanglement swapping establishes entanglement between remote quantum nodes that do not initially share an elementary entangled link. For a multi-hop candidate path, elementary entangled pairs are first generated between neighboring nodes, after which the intermediate nodes perform Bell-state measurements (BSMs) to extend entanglement from the source to the destination [6].

Let

$$P = (v_0, v_1, \ldots, v_{X(P)}) \tag{S16}$$

denote a candidate path containing $X(P)$ elementary links. The set of intermediate nodes are

$$I(P) = \{v_1, v_2, \ldots, v_{X(P)-1}\}. \tag{S17}$$

Consequently, a path containing $X(P)$ elementary links requires $X(P) - 1$ entanglement-swapping operations. For a direct path with $X(P) = 1$, no entanglement swapping is required.

In the physical-state model used in this work, the fidelity after entanglement swapping is not approximated by multiplying the elementary-link fidelities. Instead, the E2E state is constructed recursively using the density-matrix swapping operation described in the main text. For two adjacent states ρ_{AB_1} and ρ_{B_2C}, a Bell-state measurement is performed on qubits B_1 and B_2, followed by the outcome-dependent Pauli correction. Repeating this procedure over all intermediate nodes produces the final state

$$\rho_{\text{E2E}}^{(M,X(P))}. \tag{S18}$$

The final E2E fidelity and relative entropy of coherence are then evaluated from the same density matrix:

$$F_{\text{E2E}}(P, M) = \langle \Phi^+ | \rho_{\text{E2E}}^{(M,X(P))} | \Phi^+ \rangle, \tag{S19}$$

and

$$C_{\text{rel,E2E}}(P, M) = S\left[\left(\rho_{\text{E2E}}^{(M,X(P))} \right)_{\text{diag}} \right] - S\left[\rho_{\text{E2E}}^{(M,X(P))} \right]. \tag{S20}$$

The network-level swapping-success probabilities are treated separately from the density-matrix state evolution. Specifically, their cumulative effect is included in the usable path capacity $U(P)$. They are therefore not included again as an additional probabilistic waiting-time factor in the swapping-time model.

The swapping-related latency used in the routing evaluation represents the classical signaling delay associated with the selected path. Let $L_{v_k,v_{k+1}}$ denote the physical length of the elementary link between two consecutive nodes. The swapping and associated signaling time are modeled as

$$T_{\text{swap}}(P) = \frac{1}{c_{\text{fiber}}} \sum_{k=0}^{X(P)-1} L_{v_k,v_{k+1}}, \tag{S21}$$

where $c_{\text{fiber}} = 2 \times 10^8$ m/s is the propagation speed in optical fiber. This formulation treats the local BSM processing time as negligible and accounts only for the path-level propagation and signaling contribution.

In the equal-link-distance scenarios, where

$$L_{v_k,v_{k+1}} = L_{\text{link}} \qquad \forall (v_k, v_{k+1}) \in P, \tag{S22}$$

Eq. (S21) reduces to

$$T_{\text{swap}}(P) = X(P)\frac{L_{\text{link}}}{c_{\text{fiber}}}. \tag{S23}$$

Although a direct path does not require a BSM, the expression for $T_{\text{swap}}(P)$ retains the associated end-to-end signaling delay. Therefore, this term is interpreted as the swapping and associated classical signaling contribution rather than solely as the local execution time of the Bell-state measurements.

The complete processing latency for candidate path P and purification level M is consequently

$$T(P, M) = T_{\text{ent}}(P) + T_{\text{pur}}(P, M) + T_{\text{swap}}(P). \tag{S24}$$

2.5 E2E Latency and Average Latency

After the entanglement generation, purification, and swapping time components are computed, the end-to-end latency of a selected path is obtained by summing these three contributions. For a selected path P, the total latency is

$$T(P) = T_{\text{gen}}(P) + T_{\text{pur}}(P) + T_{\text{sw}}(P). \tag{S25}$$

For a request r, let $P_r^\star$ denote the selected candidate path. The latency experienced by request r is therefore [7];

$$T_r = T(P_r^\star) = T_{\text{gen}}(P_r^\star) + T_{\text{pur}}(P_r^\star) + T_{\text{sw}}(P_r^\star). \tag{S26}$$

A request is considered time-feasible only if its selected path satisfies:

$$T(P_r^\star) \leq T_{\text{th}}, \tag{S27}$$

where T_{th} is the per-request latency threshold. Therefore, requests that require more processing time than the allowed threshold are dropped.

The average latency reported in the results is computed over the set of successfully served requests. Let this set be denoted by $\mathcal{R}_{\text{succ}}$. A request belongs to $\mathcal{R}_{\text{succ}}$ only if the selected path and purification level satisfy the final QoS constraints of the corresponding routing strategy. Therefore, requests that are rejected during final validation are not included in the latency average. The average latency is defined as

$$AL = \frac{1}{|\mathcal{R}_{\text{succ}}|} \sum_{r \in \mathcal{R}_{\text{succ}}} T(P_r^\star, M_r^\star). \tag{S28}$$

Similarly, the average purification time is computed over the same set of successfully served requests:

$$APT = \frac{1}{|\mathcal{R}_{\mathrm{succ}}|} \sum_{r \in \mathcal{R}_{\mathrm{succ}}} T_{\mathrm{pur}}(P_r^{\star}, M_r^{\star}). \tag{S29}$$

The average path length is also computed over $\mathcal{R}_{\mathrm{succ}}$:

$$APL = \frac{1}{|\mathcal{R}_{\mathrm{succ}}|} \sum_{r \in \mathcal{R}_{\mathrm{succ}}} X(P_r^{\star}). \tag{S30}$$

3 Detailed Routing Algorithms

This section presents the step-by-step workflows of FARA-PostREC, FARA-NoREC, and CA-FARA algorithms. The algorithms differ mainly in how they handle purification and coherence-aware path selection.

3.1 FARA-NoREC Routing Algorithm

FARA-NoREC is a fidelity-aware routing and purification strategy in which the relative entropy of coherence is excluded from purification planning, candidate-path feasibility checking, path selection, and final request validation. Although the E2E REC value is obtained from the lookup table for performance reporting, it does not affect any routing decision.

For each incoming request $r = (s_r, t_r, d_{x,r}, F_{\mathrm{th},r}, a_r, b_r)$, the algorithm first constructs a set of edge-disjoint candidate paths between the source and destination nodes. For every candidate path P, the algorithm calculates the usable capacity, entanglement-generation time, swapping time, hop count, and normalized amplitude-damping duration τ/T_1.

The candidate purification levels $\mathcal{M} = \{0, 1, \ldots, M_{\max}\}$ are then examined in ascending order. For each purification level M, the precomputed amplitude-damping–BBPSSW lookup table returns the corresponding E2E fidelity, E2E REC, purification success information, and expected raw Bell-pair requirement. A purification level is feasible for FARA-NoREC if

$$F_{\mathrm{E2E}}(P, M) \geq F_{\mathrm{th},r}, \tag{S31}$$

$$T(P, M) \leq T_{\mathrm{th}}, \tag{S32}$$

and

$$U(P) \geq B_{\mathrm{req}}(P, M, r). \tag{S33}$$

The first purification level satisfying these constraints is selected as the minimum feasible purification level for the candidate path. Candidate paths without a feasible purification level or with a non-positive EGR are discarded. Among the remaining paths, FARA-NoREC selects the path with the highest EGR. The request is considered successful only if the selected path satisfies the final fidelity and latency constraints. No REC threshold is applied during final validation.

Algorithm S1 FARA-NoREC routing and purification procedure

Require: Quantum network $G = (V, E, C)$; request $r = (s_r, t_r, d_{x,r}, F_{\text{th},r}, a_r, b_r)$; lookup table D; maximum purification level $M_{\max}$; latency threshold T_{th}; active reservation set $\mathcal{A}_r$

Ensure: Selected path $P_r^\star$, purification level $M_r^\star$, or request rejection

Generate the edge-disjoint candidate-path set $\mathcal{P}_r$ between s_r and t_r
if $\mathcal{P}_r = \emptyset$ **then**
  return Reject(*no path*)
$\mathcal{P}_r^{\text{feasible}} \leftarrow \emptyset$
for all $P \in \mathcal{P}_r$ **do**
  Compute the hop count $X(P)$
  Compute the usable path capacity $U(P)$
  Compute $T_{\text{ent}}(P)$ and $T_{\text{swap}}(P)$
  Compute the normalized damping duration $\tau/T_1 = T_{\text{ent}}(P)/T_1$
  Determine the effective initial fidelity F_{initial}^P
  $M_P^\star \leftarrow$ undefined
  for $M = 0$ to $M_{\max}$ **do**
    Query

$$\big(F_{\text{E2E}}(P, M), C_{\text{rel,E2E}}(P, M), B_{\text{req}}(P, M, r)\big) \leftarrow D\Big(F_{\text{initial}}^P, \tau/T_1, M, X(P)\Big)$$

    Compute

$$T(P, M) = T_{\text{ent}}(P) + T_{\text{pur}}(P, M) + T_{\text{swap}}(P)$$

    if $F_{\text{E2E}}(P, M) \geq F_{\text{th},r}$ **and** $T(P, M) \leq T_{\text{th}}$ **and** $U(P) \geq B_{\text{req}}(P, M, r)$ **then**
      $M_P^\star \leftarrow M$
      break
  if $M_P^\star$ is defined **then**
    Compute EGR(P)
    if EGR$(P) > 0$ **then**
      Add $(P, M_P^\star, \text{EGR}(P))$ to $\mathcal{P}_r^{\text{feasible}}$
if $\mathcal{P}_r^{\text{feasible}} = \emptyset$ **then**
  return Reject(*no feasible path*)
Filter the feasible candidate paths according to the active reservations. A candidate path is retained only if it does not share any edge with an already reserved path whose activity interval overlaps with $[a_r, b_r]$:

$$\mathcal{P}_r^{\text{avail}} = \Big\{P \in \mathcal{P}_r^{\text{feasible}} : \nexists \hat{r} \in \mathcal{A}_r \text{ s.t. } [a_r, b_r] \cap [a_{\hat{r}}, b_{\hat{r}}] \neq \emptyset \text{ and } E(P) \cap E(P_{\hat{r}}^\star) \neq \emptyset\Big\}.$$

if $\mathcal{P}_r^{\text{avail}} = \emptyset$ **then**
  return Reject(*overlap conflict*)
Select

$$P_r^\star = \arg\max_{P \in \mathcal{P}_r^{\text{avail}}} \text{EGR}(P)$$

Set $M_r^\star \leftarrow M_{P_r^\star}^\star$
Generate elementary entanglement along $P_r^\star$
if $M_r^\star > 0$ **then**
  Apply $M_r^\star$ BBPSSW purification levels
Perform entanglement swapping at the intermediate nodes
Evaluate the final $F_{\text{E2E}}(P_r^\star)$, $C_{\text{rel,E2E}}(P_r^\star)$, and $T(P_r^\star, M_r^\star)$
if $F_{\text{E2E}}(P_r^\star) \geq F_{\text{th},r}$ **and** $T(P_r^\star, M_r^\star) \leq T_{\text{th}}$ **then**
  Add the reservation $(P_r^\star, [a_r, b_r])$ to the active reservation set
  return $\big(P_r^\star, M_r^\star, \text{Successful}\big)$
else
  return Reject(*final fidelity or latency*)

3.2 FARA-PostREC Routing Algorithm

FARA-PostREC is a fidelity-aware routing and purification strategy in which the relative entropy of coherence is excluded from purification-level determination, candidate-path feasibility checking, and path selection. However, unlike FARA-NoREC, FARA-PostREC applies the E2E REC threshold after the path and purification level have been selected. Therefore, REC acts as a post-selection reliability constraint rather than a proactive routing criterion.

For each incoming request $r = (s_r, t_r, d_{x,r}, F_{\text{th},r}, a_r, b_r)$, the algorithm first constructs a set of edge-disjoint candidate paths between the source and destination nodes. For every candidate path P, it computes the usable capacity, entanglement-generation time, swapping time, hop count, and normalized amplitude-damping duration τ/T_1.

The candidate purification levels $\mathcal{M} = \{0, 1, \ldots, M_{\max}\}$ are examined in ascending order. For each purification level M, the precomputed amplitude-damping–BBPSSW lookup table returns the corresponding E2E fidelity, E2E REC, purification information, and expected raw Bell-pair requirement. However, the REC value does not participate in purification-level feasibility. A purification level is feasible for FARA-PostREC if

$$F_{\mathrm{E2E}}(P, M) \geq F_{\mathrm{th},r}, \tag{S34}$$

$$T(P, M) \leq T_{\mathrm{th}}, \tag{S35}$$

and

$$U(P) \geq B_{\mathrm{req}}(P, M, r). \tag{S36}$$

The first purification level satisfying these constraints is selected as the minimum feasible purification level for the candidate path. Candidate paths without a feasible purification level or with a non-positive EGR are discarded. Among the remaining feasible paths, FARA-PostREC selects the path with the highest EGR.

After path selection, the final E2E fidelity, REC, and latency are evaluated. The request is considered successful only if the selected path satisfies the fidelity, latency, and REC requirements. Consequently, a path that was feasible during fidelity-based purification planning may still be rejected during the final REC validation stage.

Algorithm S2 FARA-PostREC routing and purification procedure

Require: Quantum network $G = (V, E, C)$; request $r = (s_r, t_r, d_{x,r}, F_{\mathrm{th},r}, a_r, b_r)$; lookup table D; maximum purification level $M_{\max}$; latency threshold T_{th}; REC threshold $C_{\mathrm{rel}}^{\mathrm{th}}$; active reservation set $\mathcal{A}_r$

Ensure: Selected path $P_r^\star$, purification level $M_r^\star$, or request rejection

Generate the edge-disjoint candidate-path set $\mathcal{P}_r$ between s_r and t_r
if $\mathcal{P}_r = \emptyset$ **then**
    return REJECT(*no path*)
$\mathcal{P}_r^{\mathrm{feasible}} \leftarrow \emptyset$
for all $P \in \mathcal{P}_r$ **do**
    Compute the hop count $X(P)$
    Compute the usable path capacity $U(P)$
    Compute $T_{\mathrm{ent}}(P)$ and $T_{\mathrm{swap}}(P)$
    Compute the normalized damping duration $\tau/T_1 = T_{\mathrm{ent}}(P)/T_1$
    Determine the effective initial fidelity F_{initial}^P
    $M_P^\star \leftarrow$ undefined
    for $M = 0$ to $M_{\max}$ **do**
        Query

$$\big(F_{\mathrm{E2E}}(P, M), C_{\mathrm{rel,E2E}}(P, M), B_{\mathrm{req}}(P, M, r)\big) \leftarrow D\Big(F_{\mathrm{initial}}^P, \tau/T_1, M, X(P)\Big)$$

        Compute

$$T(P, M) = T_{\mathrm{ent}}(P) + T_{\mathrm{pur}}(P, M) + T_{\mathrm{swap}}(P)$$

        if $F_{\mathrm{E2E}}(P, M) \geq F_{\mathrm{th},r}$ **and** $T(P, M) \leq T_{\mathrm{th}}$ **and** $U(P) \geq B_{\mathrm{req}}(P, M, r)$ **then**
            $M_P^\star \leftarrow M$
            break
    if $M_P^\star$ is defined **then**
        Compute $\mathrm{EGR}(P)$
        if $\mathrm{EGR}(P) > 0$ **then**
            Add $(P, M_P^\star, \mathrm{EGR}(P))$ to $\mathcal{P}_r^{\mathrm{feasible}}$
if $\mathcal{P}_r^{\mathrm{feasible}} = \emptyset$ **then**
    return REJECT(*no feasible path*)
Filter the feasible candidate paths according to the active reservations. A candidate path is retained only if it does not share any edge with an already reserved path whose activity interval overlaps with $[a_r, b_r]$:

$$\mathcal{P}_r^{\mathrm{avail}} = \Big\{P \in \mathcal{P}_r^{\mathrm{feasible}} : \nexists \hat{r} \in \mathcal{A}_r \text{ s.t. } [a_r, b_r] \cap [a_{\hat{r}}, b_{\hat{r}}] \neq \emptyset \text{ and } E(P) \cap E(P_{\hat{r}}^\star) \neq \emptyset\Big\}.$$

if $\mathcal{P}_r^{\mathrm{avail}} = \emptyset$ **then**
    return REJECT(*overlap conflict*)
Select

$$P_r^\star = \arg\max_{P \in \mathcal{P}_r^{\mathrm{avail}}} \mathrm{EGR}(P)$$

Set $M_r^\star \leftarrow M_{P_r^\star}^\star$
Generate elementary entanglement along $P_r^\star$
if $M_r^\star > 0$ **then**
    Apply $M_r^\star$ BBPSSW purification levels
Perform entanglement swapping at the intermediate nodes
Evaluate the final $F_{\mathrm{E2E}}(P_r^\star)$, $C_{\mathrm{rel,E2E}}(P_r^\star)$, and $T(P_r^\star, M_r^\star)$
if $F_{\mathrm{E2E}}(P_r^\star) \geq F_{\mathrm{th},r}$ **and** $T(P_r^\star, M_r^\star) \leq T_{\mathrm{th}}$ **and** $C_{\mathrm{rel,E2E}}(P_r^\star) \geq C_{\mathrm{rel}}^{\mathrm{th}}$ **then**
    Add the reservation $(P_r^\star, [a_r, b_r])$ to the active reservation set
    return $\big(P_r^\star, M_r^\star, \text{SUCCESSFUL}\big)$
else
    return REJECT(*final fidelity, latency, or REC*)

3.3 CAFARA Routing Algorithm

CAFARA is a coherence- and fidelity-aware routing and purification strategy that incorporates the end-to-end relative entropy of coherence directly into purification-level determination, candidate-path feasibility checking, path selection, and final request validation. Unlike FARA-PostREC, which evaluates REC only after selecting a path, CAFARA proactively excludes purification levels and candidate paths that cannot jointly satisfy the requested fidelity and REC

requirements.

For each incoming request $r = (s_r, t_r, d_{x,r}, F_{\text{th},r}, a_r, b_r)$, CAFARA first constructs a set of edge-disjoint candidate paths between the source and destination nodes. For every candidate path P, the algorithm computes the usable capacity, entanglement-generation time, swapping time, hop count, and normalized amplitude-damping duration τ/T_1.

The candidate purification levels $\mathcal{M} = \{0, 1, \ldots, M_{\max}\}$ are examined in ascending order. For each purification level M, the precomputed amplitude-damping–BBPSSW lookup table returns the corresponding E2E fidelity, E2E REC, purification information, and expected raw Bell-pair requirement. A purification level is feasible for CAFARA only if

$$F_{\text{E2E}}(P, M) \geq F_{\text{th},r}, \tag{S37}$$

$$C_{\text{rel,E2E}}(P, M) \geq C_{\text{rel}}^{\text{th}}, \tag{S38}$$

$$T(P, M) \leq T_{\text{th}}, \tag{S39}$$

and

$$U(P) \geq B_{\text{req}}(P, M, r). \tag{S40}$$

The first purification level satisfying all four constraints is selected as the minimum feasible purification level for the candidate path. Therefore, CAFARA avoids unnecessary purification while ensuring that both the fidelity and coherence requirements are satisfied. A higher purification level is selected only when the requested fidelity has already been achieved, but the corresponding E2E REC remains below the required threshold.

Candidate paths for which no jointly feasible purification level exists are discarded. Paths with a non-positive entanglement generation rate are also removed. Among the remaining candidate paths, CAFARA selects the path having the highest EGR. The selected path is subsequently processed through entanglement generation, BBPSSW purification, and entanglement swapping. A request is counted as successful only if the final state satisfies the fidelity, REC, latency, and capacity requirements.

Algorithm S3 CAFARA routing and purification procedure

Require: Quantum network $G = (V, E, C)$; request $r = (s_r, t_r, d_{x,r}, F_{\text{th},r}, a_r, b_r)$; lookup table D; maximum purification level $M_{\max}$; latency threshold T_{th}; REC threshold $C_{\text{rel}}^{\text{th}}$; active reservation set $\mathcal{A}_r$

Ensure: Selected path $P_r^\star$, purification level $M_r^\star$, or request rejection

Generate the edge-disjoint candidate-path set $\mathcal{P}_r$ between s_r and t_r
if $\mathcal{P}_r = \emptyset$ **then**
  return REJECT(*no path*)
$\mathcal{P}_r^{\text{feasible}} \leftarrow \emptyset$
for all $P \in \mathcal{P}_r$ **do**
  Compute the hop count $X(P)$
  Compute the usable path capacity $U(P)$
  Compute $T_{\text{ent}}(P)$ and $T_{\text{swap}}(P)$
  Compute the normalized damping duration $\tau/T_1 = T_{\text{ent}}(P)/T_1$
  Determine the effective initial fidelity F_{initial}^P
  $M_P^\star \leftarrow$ undefined
  for $M = 0$ to $M_{\max}$ **do**
    Query

$$\big(F_{\text{E2E}}(P, M), C_{\text{rel,E2E}}(P, M), B_{\text{req}}(P, M, r)\big) \leftarrow D\Big(F_{\text{initial}}^P, \tau/T_1, M, X(P)\Big)$$

    Compute

$$T(P, M) = T_{\text{ent}}(P) + T_{\text{pur}}(P, M) + T_{\text{swap}}(P)$$

    if $F_{\text{E2E}}(P, M) \geq F_{\text{th},r}$ **and** $C_{\text{rel,E2E}}(P, M) \geq C_{\text{rel}}^{\text{th}}$ **and** $T(P, M) \leq T_{\text{th}}$ **and** $U(P) \geq B_{\text{req}}(P, M, r)$ **then**
      $M_P^\star \leftarrow M$
      break
  if $M_P^\star$ is defined **then**
    Compute EGR(P)
    if EGR$(P) > 0$ **then**
      Add $(P, M_P^\star, \text{EGR}(P))$ to $\mathcal{P}_r^{\text{feasible}}$
if $\mathcal{P}_r^{\text{feasible}} = \emptyset$ **then**
  return REJECT(*no jointly feasible path*)
Filter the jointly feasible candidate paths according to the active reservations. A candidate path is retained only if it does not share any edge with an already reserved path whose activity interval overlaps with $[a_r, b_r]$:

$$\mathcal{P}_r^{\text{avail}} = \Big\{P \in \mathcal{P}_r^{\text{feasible}} : \nexists \hat{r} \in \mathcal{A}_r \text{ s.t. } [a_r, b_r] \cap [a_{\hat{r}}, b_{\hat{r}}] \neq \emptyset \text{ and } \text{Edges}(P) \cap \text{Edges}(P_{\hat{r}}^\star) \neq \emptyset\Big\}.$$

if $\mathcal{P}_r^{\text{avail}} = \emptyset$ **then**
  return REJECT(*overlap conflict*)
Select

$$P_r^\star = \arg\max_{P \in \mathcal{P}_r^{\text{avail}}} \text{EGR}(P)$$

Set $M_r^\star \leftarrow M_{P_r^\star}^\star$
Generate elementary entanglement along $P_r^\star$
if $M_r^\star > 0$ **then**
  Apply $M_r^\star$ BBPSSW purification levels
Perform entanglement swapping at the intermediate nodes
Evaluate the final $F_{\text{E2E}}(P_r^\star)$, $C_{\text{rel,E2E}}(P_r^\star)$, and $T(P_r^\star, M_r^\star)$
if $F_{\text{E2E}}(P_r^\star) \geq F_{\text{th},r}$ **and** $C_{\text{rel,E2E}}(P_r^\star) \geq C_{\text{rel}}^{\text{th}}$ **and** $T(P_r^\star, M_r^\star) \leq T_{\text{th}}$ **and** $U(P_r^\star) \geq B_{\text{req}}(P_r^\star, M_r^\star, r)$ **then**
  Add the reservation $(P_r^\star, [a_r, b_r])$ to the active reservation set
  return $\big(P_r^\star, M_r^\star, \text{SUCCESSFUL}\big)$
else
  return REJECT(*final quality, latency, or capacity*)

4 Time Complexity Analysis

This section analyzes the time complexity of the routing algorithms that are proposed in the article "Coherence and Fidelity Aware Routing in Quantum Networks." The analysis excludes topology generation, since the network graph is assumed to be given before request processing

starts. The online computational complexity is expressed in terms of the total number of requests R, the graph size $(N, |E|)$, the number of candidate paths n_r generated for each request, the maximum candidate-path length H_r, the number of examined purification levels $M_{\max} + 1$, and the number of entries K searched during one lookup-table query.

4.1 Time Complexity of the FARA-NoREC Routing Algorithm

This subsection analyzes the worst-case online computational complexity of the FARA-NoREC routing and purification procedure presented in Algorithm S1. The offline construction of the amplitude-damping–BBPSSW lookup table D is excluded because it is performed once before online request processing. However, the cost of querying and filtering the precomputed lookup table during routing is included.

Let R denote the total number of connection requests, $N = |V|$ the number of quantum nodes, and $|E|$ the number of quantum links. For request r, let

$$n_r = |\mathcal{P}_r| \tag{S41}$$

denote the number of generated edge-disjoint candidate paths, and let H_r denote the maximum hop count among these paths. For a request having no candidate path, H_r is taken as zero. The algorithm examines at most $M_{\max} + 1$ purification levels for each candidate path. The analysis is decomposed into candidate-path generation, path-level metric computation, purification-level search, EGR evaluation, overlap-aware path selection, and final request validation.

Edge-disjoint candidate-path generation. For each request r, FARA-NoREC constructs the candidate-path set $\mathcal{P}_r$ using a greedy repeated-shortest-path procedure. The network graph is first copied, and an unweighted shortest path between s_r and t_r is computed. The edges of the identified path are then removed from the copied graph, and the shortest-path search is repeated until no further source–destination path exists.

Copying the graph requires $\mathcal{O}(N + |E|)$ time [8]. The procedure then performs n_r successful breadth-first searches to generate the candidate paths and one additional unsuccessful search to terminate the loop. Since each breadth-first search has worst-case complexity $\mathcal{O}(N + |E|)$, the total search cost is $\mathcal{O}((n_r + 1)(N + |E|))$ [8]. Moreover, because the generated paths are edge-disjoint, at most $|E|$ edges are removed over all iterations. Consequently,

$$\begin{aligned} T_{\text{path}}(r) &= \mathcal{O}(N + |E| + (n_r + 1)(N + |E|) + |E|) \\ &= \mathcal{O}((n_r + 1)(N + |E|)) \,. \end{aligned} \tag{S42}$$

Using the general upper bound $n_r \le |E|$, this can also be written as

$$T_{\text{path}}(r) = \mathcal{O}(|E|(N + |E|)) \,. \tag{S43}$$

Path-level metric computation. A candidate path $P \in \mathcal{P}_r$ is represented as

$$P = (v_0, v_1, \ldots, v_{H(P)}), \tag{S44}$$

where $v_0 = s_r$, $v_{H(P)} = t_r$, and

$$H(P) = |P| - 1 \tag{S45}$$

is the number of hops, equivalently the number of elementary links, in the path. The routing algorithm denotes the same quantity by $X(P)$; hence,

$$X(P) = H(P). \tag{S46}$$

For request r, define

$$H_r = \max_{P \in \mathcal{P}_r} H(P). \tag{S47}$$

The hop count is obtained from the length of the stored path list and therefore requires

$$T_X(P) = \mathcal{O}(1). \tag{S48}$$

The usable capacity is evaluated as

$$U(P) = \left(\min_{0 \le k < H(P)} c(v_k, v_{k+1}) \right) \prod_{u \in I(P)} p_{\text{swap}}(u), \tag{S49}$$

where

$$I(P) = \{v_1, v_2, \dots, v_{H(P)-1}\} \tag{S50}$$

is the set of intermediate nodes, $c(v_k, v_{k+1})$ is the currently available link capacity, and $p_{\text{swap}}(u)$ is the swapping-success probability of intermediate node u.

Computing the swapping-success product requires traversing at most $H(P) - 1$ intermediate nodes, while finding the bottleneck capacity requires examining the $H(P)$ links. Let $T_U(P)$ denote the computational time required to evaluate $U(P)$. Then,

$$T_U(P) = \mathcal{O}(H(P)) = \mathcal{O}(H_r). \tag{S51}$$

Entanglement-generation, swapping-time, and state-parameter computation. For each candidate path P, FARA-NoREC computes the physical entanglement-generation time $T_{\text{ent}}(P)$, the swapping-related signaling time $T_{\text{swap}}(P)$, the normalized damping duration τ/T_1, and the effective initial fidelity F^P_{initial}.

The entanglement-generation-time calculation traverses all $H(P)$ links. For each link, it performs a constant number of graph-attribute accesses and arithmetic operations. Therefore,

$$T^{\text{comp}}_{\text{ent}}(P) = \mathcal{O}(H(P)), \tag{S52}$$

where $T^{\text{comp}}_{\text{ent}}(P)$ denotes the computational cost of evaluating the physical quantity $T_{\text{ent}}(P)$.

The swapping-time calculation visits the $H(P) - 1$ intermediate nodes. At each node, it retrieves adjacent-link lengths, selects a reference length, and performs a constant number of arithmetic operations. Hence,

$$T^{\text{comp}}_{\text{swap}}(P) = \mathcal{O}(H(P)). \tag{S53}$$

Once $T_{\text{ent}}(P)$ has been obtained, calculating

$$\frac{\tau}{T_1} = \frac{T_{\text{ent}}(P)}{T_1} \tag{S54}$$

requires one division and therefore has complexity $\mathcal{O}(1)$.

Since the elementary links may have different initial fidelities, the effective path-level initial fidelity is

$$F^P_{\text{initial}} = \min_{\ell \in P} F^{(\ell)}_{\text{initial}}. \tag{S55}$$

Determining this minimum requires examining all $H(P)$ links and therefore has complexity $\mathcal{O}(H(P))$.

Consequently, the combined computational cost of these path-level operations is

$$T^{\text{comp}}_{\text{state}}(P) = \mathcal{O}(H(P)). \tag{S56}$$

Purification-level search and lookup-table evaluation. For each candidate path P, FARA-NoREC examines

$$\mathcal{M} = \{0, 1, \ldots, M_{\max}\} \tag{S57}$$

in ascending order and selects the first purification level satisfying the fidelity, capacity, and latency constraints.

Let $|D|$ denote the total number of rows in the complete lookup table. Before examining the purification levels, the implementation extracts the available source-fidelity values, identifies the value nearest to F^P_{initial}, and constructs the corresponding source-fidelity subset. Processing the complete lookup table and obtaining the unique source-fidelity values has worst-case complexity

$$T_{\text{pre}}(D) = \mathcal{O}(|D| \log |D|) \,. \tag{S58}$$

Let K denote the number of rows in the selected source-fidelity subset, and let $K_{M,X}$ denote the number of rows corresponding to one fixed purification level M and hop count $X(P)$. For each purification level, the lookup function filters the K rows according to M and $X(P)$, requiring $\mathcal{O}(K)$ time. It then sorts the resulting $K_{M,X}$ rows according to τ/T_1, requiring $\mathcal{O}(K_{M,X} \log K_{M,X})$ time. The exact-grid search and interpolation-interval identification require at most $\mathcal{O}(K_{M,X})$ additional time. Thus, one lookup query has complexity

$$T_D(K, K_{M,X}) = \mathcal{O}(K + K_{M,X} \log K_{M,X}) \,. \tag{S59}$$

Since $K_{M,X} \leq K$, a simpler worst-case upper bound is

$$T_D(K) = \mathcal{O}(K \log K) \,. \tag{S60}$$

Purification-time computation. For each candidate purification level M, FARA-NoREC evaluates the physical purification latency $T_{\text{pur}}(P, M)$. When $M = 0$, the function returns immediately and has complexity $\mathcal{O}(1)$.

For $M > 0$, the implementation traverses the $H(P)$ elementary links. For each link, it retrieves the physical distance, computes the round-trip classical signaling time, adds the local-operation contribution, and multiplies the resulting per-round latency by M. The resulting link-level values are either maximized under parallel execution or summed under sequential execution. Both operations require a linear traversal of the link values. Therefore,

$$T^{\text{comp}}_{\text{pur}}(P, M) = \mathcal{O}(H(P)) \,. \tag{S61}$$

Although the physical purification latency increases with M, its computational complexity does not contain an additional multiplicative factor of M, because the implementation uses a single multiplication instead of explicitly simulating the purification rounds.

For each M, the remaining calculations, including total latency, raw Bell-pair demand, and feasibility comparisons, require constant time. Since at most $M_{\max} + 1$ levels are examined, the worst-case purification-level search complexity is

$$T_{\text{search}}(P) = \mathcal{O}((M_{\max} + 1) \, [K \log K + H(P)]) \,. \tag{S62}$$

If no feasible level is found, at most $M_{\max} + 1$ stored candidate records are scanned to identify the rejection reason. This does not change the dominant asymptotic order. Including source-fidelity preprocessing, the total purification-level determination complexity is therefore

$$T_M(P) = \mathcal{O}(|D| \log |D| + (M_{\max} + 1) \, [K \log K + H(P)]) \,. \tag{S63}$$

Entanglement-generation-rate computation. If a feasible purification level exists for candidate path P, FARA-NoREC computes its path-level entanglement generation rate $\mathrm{EGR}(P)$.

Constructing the link-parameter list and evaluating the link-level generation statistics each require $\mathcal{O}(H(P))$ time. The expected E2E generation time and minimum required storage time are computed by the recursive functions `T_route` and `tau_route`. For a route segment containing h links, each function satisfies

$$T_{\mathrm{rec}}(h) = 2T_{\mathrm{rec}}\left(\frac{h}{2}\right) + \mathcal{O}(h), \tag{S64}$$

because the route is divided into two subsegments and the classical communication delays are summed at each recursion level.

The recurrence can be explained using a recursion-tree argument. At each recursion level, the route is divided into smaller subsegments, but the total amount of work over all subsegments remains linear in h. Since the segment length is halved at every recursive step, the recursion depth is $\log h$. Therefore, the total cost is $\mathcal{O}(h)$ work per level over $\log h$ levels, which gives

$$T_{\mathrm{rec}}(h) = \mathcal{O}(h \log h). \tag{S65}$$

The final route-feasibility and rate calculation scans the link-level storage margins once and requires $\mathcal{O}(H(P))$ time. Consequently,

$$T_{\mathrm{EGR}}^{\mathrm{comp}}(P) = \mathcal{O}\big(H(P) \log\big(H(P) + 1\big)\big). \tag{S66}$$

Since $H(P) \leq H_r$, the corresponding upper bound is

$$T_{\mathrm{EGR}}^{\mathrm{comp}}(P) = \mathcal{O}(H_r \log(H_r + 1)). \tag{S67}$$

Feasible-path filtering, overlap checking, and maximum-EGR path selection. Let

$$f_r = \left|\mathcal{P}_r^{\mathrm{feasible}}\right| \tag{S68}$$

denote the number of feasible candidate paths, where $f_r \leq n_r$. Let $\mathcal{A}_r$ denote the active-reservation set considered for request r, and let

$$A_r = |\mathcal{A}_r|. \tag{S69}$$

For each feasible candidate path, constructing or retrieving its edge set requires at most $\mathcal{O}(H_r)$ time. The temporal-overlap test with one active reservation requires constant time. When the intervals overlap, checking whether the corresponding paths share an edge requires at most $\mathcal{O}(H_r)$ time.

In the worst case, each of the f_r paths is compared with all A_r active reservations. Including the construction or retrieval of the candidate edge set, the overlap-checking cost is

$$T_{\mathrm{ov}}(r) = \mathcal{O}(f_r(A_r + 1)H_r). \tag{S70}$$

After conflicting paths have been removed, the remaining candidates are scanned once to identify the path having the maximum positive EGR. This scan requires $\mathcal{O}(f_r)$ time and is dominated by the overlap-checking term. Therefore,

$$T_{\mathrm{select}}(r) = \mathcal{O}(f_r(A_r + 1)H_r). \tag{S71}$$

Since $f_r \leq n_r$,

$$T_{\mathrm{select}}(r) = \mathcal{O}(n_r(A_r + 1)H_r). \tag{S72}$$

Selected-path validity and final request validation. After selecting the maximum-EGR path $P_r^\star$, FARA-NoREC checks whether all its elementary links remain valid. Let $H(P_r^\star)$ denote the hop count of the selected path. In the worst case, all links must be examined; hence,

$$T_{\text{valid}}(r) = \mathcal{O}(H(P_r^\star)) = \mathcal{O}(H_r). \tag{S73}$$

FARA-NoREC then evaluates

$$T(P_r^\star, M_r^\star) \leq T_{\text{th}} \tag{S74}$$

and

$$F_{\text{E2E}}(P_r^\star, M_r^\star) \geq F_{\text{th},r}. \tag{S75}$$

Since these values have already been computed and stored, the final success decision requires a constant number of accesses and comparisons:

$$T_{\text{final}}(r) = \mathcal{O}(1). \tag{S76}$$

Thus, the combined selected-path validity and final-validation cost is

$$T_{\text{post}}(r) = \mathcal{O}(H_r). \tag{S77}$$

Overall time complexity of FARA-NoREC. For request r, let K_r denote the maximum size of a source-fidelity lookup subset used while processing its candidate paths. Combining candidate-path generation, candidate evaluation, EGR calculation, overlap-aware selection, and final validation gives

$$\begin{aligned} T_{\text{FARA-NoREC}}(r) = \mathcal{O}\Big((n_r+1)(N+|E|) + n_r\big[|D|\log|D| + (M_{\max}+1)(K_r\log K_r + H_r) \\ + H_r\log(H_r+1) + (A_r+1)H_r\big]\Big). \end{aligned} \tag{S78}$$

The separate $\mathcal{O}(n_r)$ maximum-EGR scan and $\mathcal{O}(H_r)$ final-validation cost are absorbed by the displayed terms.

For a workload containing R requests, define

$$n_{\max} = \max_{1\leq r\leq R} n_r, \qquad H_{\max} = \max_{1\leq r\leq R} H_r, \tag{S79}$$

and

$$A_{\max} = \max_{1\leq r\leq R} A_r, \qquad K_{\max} = \max_{1\leq r\leq R} K_r. \tag{S80}$$

The total worst-case online complexity is

$$\begin{aligned} T_{\text{FARA-NoREC}} = \mathcal{O}\Big(R\Big[(n_{\max}+1)(N+|E|) + n_{\max}\big(|D|\log|D| + (M_{\max}+1)(K_{\max}\log K_{\max} + H_{\max}) \\ + H_{\max}\log(H_{\max}+1) + (A_{\max}+1)H_{\max}\big)\Big]\Big). \end{aligned} \tag{S81}$$

The physical entanglement-generation, purification, and swapping durations are not computational-complexity terms themselves. The analysis includes only the computational work required to evaluate these physical latency quantities.

4.2 Time Complexity of the FARA-PostREC Routing Algorithm

FARA-PostREC follows the same candidate-path generation, path-level metric computation, purification-level search, EGR evaluation, overlap-aware path selection, and selected-path validation procedures as FARA-NoREC. Therefore, the complexity results derived in Section 4.1 also apply to FARA-PostREC.

In both FARA variants, the purification level is selected using the fidelity, capacity, and latency constraints; the REC threshold does not participate in purification-level determination or path selection. The difference is that FARA-PostREC additionally applies the final coherence condition

$$C_{\text{rel,E2E}}\left(P_r^{\star}, M_r^{\star}\right) \geq C_{\text{rel}}^{\text{th}} \tag{S82}$$

after the path $P_r^{\star}$ and purification level $M_r^{\star}$ have been selected.

The final E2E REC value is already retrieved from the lookup table and stored during candidate-path evaluation. Consequently, the additional post-selection REC validation requires only one value access and one comparison, giving

$$T_{\text{REC-check}}(r) = \mathcal{O}(1). \tag{S83}$$

Thus, the final validation cost of FARA-PostREC remains

$$T_{\text{final}}^{\text{PostREC}}(r) = \mathcal{O}(1), \tag{S84}$$

and the combined selected-path validity and final-validation cost remains $\mathcal{O}(H_r)$.

Accordingly, the worst-case online complexity for processing request r is

$$\begin{aligned} T_{\text{FARA-PostREC}}(r) = \mathcal{O}\Big((n_r+1)(N+|E|) + n_r\big[|D|\log|D| + (M_{\max}+1)(K_r\log K_r + H_r) \\ + H_r\log(H_r+1) + (A_r+1)H_r\big]\Big). \end{aligned} \tag{S85}$$

For a workload containing R requests, the total worst-case online complexity is

$$\begin{aligned} T_{\text{FARA-PostREC}} = \mathcal{O}\Big(R\Big[(n_{\max}+1)(N+|E|) + n_{\max}\big(|D|\log|D| + (M_{\max}+1)(K_{\max}\log K_{\max} + H_{\max}) \\ + H_{\max}\log(H_{\max}+1) + (A_{\max}+1)H_{\max}\big)\Big]\Big). \end{aligned} \tag{S86}$$

Therefore, FARA-PostREC and FARA-NoREC have the same asymptotic time complexity. The additional final REC comparison in FARA-PostREC changes only the constant computational factor and not the Big-$\mathcal{O}$ order.

4.3 Time Complexity of the CAFARA Routing Algorithm

CAFARA follows the same candidate-path generation, path-level metric computation, lookup-table evaluation, purification-time calculation, EGR computation, overlap-aware path selection, and selected-path validation procedures analyzed for FARA-NoREC in Section 4.1. The principal computational difference is that CAFARA includes the E2E REC constraint directly in the purification-level feasibility test.

For every candidate path P and purification level M, CAFARA evaluates

$$C_{\text{rel,E2E}}(P, M) \geq C_{\text{rel}}^{\text{th}} \tag{S87}$$

in addition to the fidelity, capacity, and latency constraints. The E2E REC value is returned together with the E2E fidelity by the same lookup-table query. Therefore, the coherence-aware feasibility test requires only one additional value access and comparison:

$$T_{\text{REC-check}}(P, M) = \mathcal{O}(1). \tag{S88}$$

Since this constant-time comparison is performed for at most $M_{\max} + 1$ purification levels, its total contribution for one candidate path is

$$\mathcal{O}(M_{\max} + 1). \tag{S89}$$

This term is already dominated by the lookup-table and purification-time computations performed during the purification-level search. Consequently, the worst-case purification-level determination complexity of CAFARA remains

$$T_M^{\text{CAFARA}}(P) = \mathcal{O}(|D| \log |D| + (M_{\max} + 1)\left[K \log K + H(P)\right]). \tag{S90}$$

CAFARA also applies the final REC condition

$$C_{\text{rel,E2E}}\left(P_r^\star, M_r^\star\right) \geq C_{\text{rel}}^{\text{th}} \tag{S91}$$

together with the final fidelity and latency requirements. Since the final REC value has already been computed and stored, this additional validation requires $\mathcal{O}(1)$ time. Therefore, the combined selected-path validity and final-validation complexity remains

$$T_{\text{post}}^{\text{CAFARA}}(r) = \mathcal{O}(H_r). \tag{S92}$$

For request r, the worst-case online complexity of CAFARA is therefore

$$\begin{aligned} T_{\text{CAFARA}}(r) = \mathcal{O}\Big((n_r + 1)(N + |E|) + n_r\big[|D| \log |D| + (M_{\max} + 1)(K_r \log K_r + H_r) \\ + H_r \log(H_r + 1) + (A_r + 1)H_r\big]\Big). \end{aligned} \tag{S93}$$

For a workload containing R requests, the total worst-case online complexity is

$$\begin{aligned} T_{\text{CAFARA}} = \mathcal{O}\Big(R\Big[(n_{\max} + 1)(N + |E|) + n_{\max}\big(|D| \log |D| + (M_{\max} + 1)(K_{\max} \log K_{\max} + H_{\max}) \\ + H_{\max} \log(H_{\max} + 1) + (A_{\max} + 1)H_{\max}\big)\Big]\Big). \end{aligned} \tag{S94}$$

Thus, CAFARA, FARA-PostREC, and FARA-NoREC have the same asymptotic worst-case time complexity. CAFARA performs an additional REC comparison for every examined purification level, whereas FARA-PostREC performs the REC comparison only during final validation and FARA-NoREC does not impose an REC condition. These differences affect the constant computational factors and the practical number of examined purification levels, but they do not change the asymptotic Big-$\mathcal{O}$ order.

References

[1] Charles H Bennett, Gilles Brassard, Sandu Popescu, Benjamin Schumacher, John A Smolin, and William K Wootters. Purification of noisy entanglement and faithful teleportation via noisy channels. *Physical review letters*, 76(5):722, 1996.

[2] H-J Briegel, Wolfgang Dür, Juan I Cirac, and Peter Zoller. Quantum repeaters: the role of imperfect local operations in quantum communication. *Physical Review Letters*, 81(26):5932, 1998.

[3] Nadja K Bernardes, Ludmiła Praxmeyer, and Peter van Loock. Rate analysis for a hybrid quantum repeater. *Physical Review A—Atomic, Molecular, and Optical Physics*, 83(1):012323, 2011.

[4] Xiao-Min Hu, Cen-Xiao Huang, Yu-Bo Sheng, Lan Zhou, Bi-Heng Liu, Yu Guo, Chao Zhang, Wen-Bo Xing, Yun-Feng Huang, Chuan-Feng Li, et al. Long-distance entanglement purification for quantum communication. *Physical review letters*, 126(1):010503, 2021.

[5] Wolfgang Dür and Hans J Briegel. Entanglement purification and quantum error correction. *Reports on Progress in Physics*, 70(8):1381–1424, 2007.

[6] Rodney Van Meter. *Quantum networking.* John Wiley & Sons, 2014.

[7] Hilal Sultan Duranoglu Tunc, Milad Ghadimi, Riccardo Bassoli, and HP Frank Fitzek. Routing in memoryless quantum networks: A lexicographic approach with fidelity guarantee. In *2025 IEEE Symposium on Computers and Communications (ISCC)*, pages 1–6. IEEE, 2025.

[8] Thomas H Cormen, Charles E Leiserson, Ronald L Rivest, and Clifford Stein. *Introduction to algorithms.* MIT press, 2022.
